# Immune microniches shape intestinal T_reg_ function

**DOI:** 10.1038/s41586-024-07251-0

**Published:** 2024-04-03

**Authors:** Yisu Gu, Raquel Bartolomé-Casado, Chuan Xu, Alice Bertocchi, Alina Janney, Cornelia Heuberger, Claire F. Pearson, Sarah A. Teichmann, Emily E. Thornton, Fiona Powrie

**Affiliations:** 1 Kennedy Institute of Rheumatology, NDORMS, University of Oxford, Oxford, UK; 2 Wellcome Sanger Institute, Wellcome Genome Campus, Hinxton, Cambridge, UK; 3 Department of Pathology, Oslo University Hospital–Rikshospitalet, Oslo, Norway; 4 Theory of Condensed Matter, Cavendish Laboratory, Department of Physics, University of Cambridge, Cambridge, UK; 5 MRC Translational Immune Discovery Unit, Weatherall Institute of Molecular Medicine, University of Oxford, Oxford, UK; 6 Nuffield Department of Medicine, University of Oxford, Oxford, UK; 7 Present Address: Roche Innovation Center Zurich, Pharma Research and Early Development, F. Hoffmann-La Roche, Schlieren, Switzerland

**Keywords:** Mucosal immunology, Cellular signalling networks, Peripheral tolerance, Lymphocyte differentiation, Imaging the immune system

## Abstract

The intestinal immune system is highly adapted to maintaining tolerance to the commensal microbiota and self-antigens while defending against invading pathogens^1,2^. Recognizing how the diverse network of local cells establish homeostasis and maintains it in the complex immune environment of the gut is critical to understanding how tolerance can be re-established following dysfunction, such as in inflammatory disorders. Although cell and molecular interactions that control T regulatory (T_reg_) cell development and function have been identified^3,4^, less is known about the cellular neighbourhoods and spatial compartmentalization that shapes microorganism-reactive T_reg_ cell function. Here we used in vivo live imaging, photo-activation-guided single-cell RNA sequencing^5–7^ and spatial transcriptomics to follow the natural history of T cells that are reactive towards *Helicobacter hepaticus* through space and time in the settings of tolerance and inflammation. Although antigen stimulation can occur anywhere in the tissue, the lamina propria—but not embedded lymphoid aggregates—is the key microniche that supports effector T_reg_ (eT_reg_) cell function. eT_reg_ cells are stable once their niche is established; however, unleashing inflammation breaks down compartmentalization, leading to dominance of CD103^+^SIRPα^+^ dendritic cells in the lamina propria. We identify and validate the putative tolerogenic interaction between CD206^+^ macrophages and eT_reg_ cells in the lamina propria and identify receptor–ligand pairs that are likely to govern the interaction. Our results reveal a spatial mechanism of tolerance in the lamina propria and demonstrate how knowledge of local interactions may contribute to the next generation of tolerance-inducing therapies.

## Abstract (web summary)

Studies in mice show that effector T regulatory cells in the gut are most functional in the lamina propria, but this homeostatic niche is disrupted in inflammation, suggesting a spatial mechanism of tolerance to commensal microorganisms.

## Main

The intestinal immune system interacts with large numbers of diverse microorganisms. This adaptive interplay between host and microorganism provides a window into how immune tolerance is established and maintained in a complex environment. The pathobiont *Helicobacter hepaticus* (*Hh*) establishes lifelong infection in the caecum of wild-type mice. A key host adaptation to ensure immune homeostasis in the face of chronic infection is the production of IL-10 by T_reg_ cells^8–10^. Colonization of previously uninfected mice is used as a model system to study how tolerance is established. Maladaptation of this response results in colitis, and similar processes underlie very early onset inflammatory bowel disease, with deficiencies in the IL-10 pathway as a major cause^11^. The mesenteric lymph nodes (MLN) have been shown to be a key site of T_reg_ cell induction^12–14^, but the key anatomical location for induction and maintenance of eT_reg_ cell suppressor function is yet to be identified. We used T cell receptor (TCR) transgenic HH7-2tg cells^15^ (TCR^*Hh*^) as sentinels in *Hh-*colonized hosts to follow the natural history of antigen-specific T cells experiencing key interactions and gaining and sustaining T_reg_ cell effector functions in the tissue microenvironment.

## eT_reg_ cells function in intestinal tissue

**Fig. 1:**
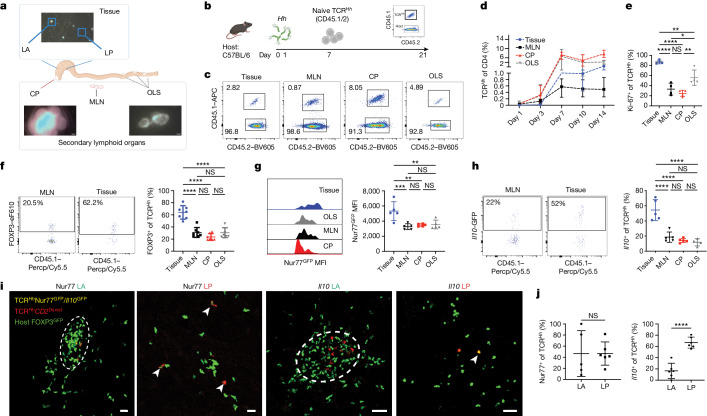
The LP niche supports the highest production of IL-10 in response to *Hh* colonization. **a**, Diagram of tissue and lymphoid structures in the mouse gut, with representative images of CP, organized lymphoid structures and LAs in *CD2*^DsRed^ mouse. Scale bars, 1,000 μm. **b**, Schematic of transfer of naive TCR^*Hh*^ T cells into *Hh*-colonized hosts. **c**, Representative fluorescence-activated cell sorting (FACS) plots of TCR^*Hh*^ T cells at the indicated sites 14 days after transfer of naive TCR^*Hh*^ T cells. **d**, Frequency of TCR^*Hh*^ cells among total CD4 T cells at indicated timepoints after transfer of naive TCR^*Hh*^ T cells. **e**, Frequency of Ki-67^+^ TCR^*Hh*^ cells at the indicated sites 14 days after transfer of naive TCR^*Hh*^ T cells. **f**, Representative FACS plots (left) and frequency of T_reg_ cells of TCR^*Hh*^ (right) at 14 days after transfer of naive TCR^*Hh*^ T cells. **g**, Representative histogram overlay (left) and GFP MFI of TCR^*Hh*^Nur77^GFP^ T cells (right) 11 days after transfer of naive TCR^*Hh*^ T cells. **h**, Representative FACS plots (left) and frequency of *Il10*^+^ cells among TCR^*Hh*^ T cells (right) 11 days after transfer of naive TCR^*Hh*^ T cells. **i**, Representative images of TCR^*Hh*^Nur77^GFP^ (left and centre left) and TCR^*Hh*^*Il10*^*GFP*^ (centre right and right) T cells in the LA and LP. TCR^*Hh*^Nur77^GFP^ and *Il10*-GFP-positive cells are indicated by white arrowheads. Scale bars, 50 μm. **j**, Frequency of Nur77^GFP^ (left) and *Il10*-GFP-positive (right) cells among TCR^*Hh*^ T cells in the LA and LP. **a**, Representative images from two individual mice over two independent imaging experiments. **c**–**e**, Four individual mice representative of two independent experiments. **f**, Seven individual mice representative of two independent experiments. **g**,**h**, Five individual mice representative of two independent experiments. **i**,**j**, Representative images and combined data from six individual mice over two independent imaging experiments. Scale bars, 50 μm. **e**–**h**, One-way ANOVA using Tukey’s multiple comparisons test. **j**, Two-tailed *t-*test. Source Data

**Extended Data Fig. 1:**
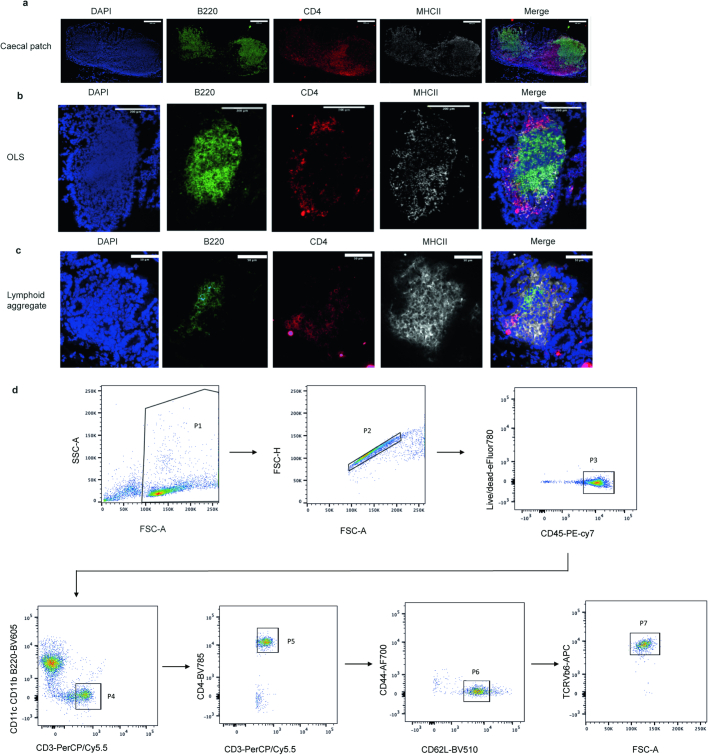
Immunofluorescent characterisation of intestinal lymphoid structures. Immunofluorescent expression of DAPI, B220, CD4 and MHCII in the a. caecal patch (scale bar 400 mm). b. organised lymphoid structure (scale bar 200 mm). c. lymphoid aggregate (scale bar 50 mm). d. Gating strategy of FACS sorting of naive T cells from TCR^Hh^ donor mice. a-c: Representative images from 2 individual mice over two independent imaging experiments.

To map where adaptive responses to *Hh* occur, we characterized the intestinal lymphoid and non-lymphoid tissue compartments of wild-type mice. Lymphoid tissue comprised secondary lymphoid organs, including MLN, the caecal patch (CP) and distal colon organized lymphoid structures (OLS) (Fig. 1a). Small lymphoid aggregates (LAs) are present in the caecum and proximal colon. In line with previous studies^16,17^, LAs do not have organized T cell and B cell zones and contain a spectrum of tissue organization from cell aggregates to cryptopatches and isolated lymphoid follicles (Extended Data Fig. 1a–c). Previous tracking of naive TCR^*Hh*^ T cells in vivo showed that they differentiated to RORγt^+^FOXP3^+^ T_reg_ cells in the colon, but that work did not exclude OLS from the analysis^15^. We sought to understand the natural history of TCR^*Hh*^ T cells in the context of the lymphoid and non-lymphoid compartments. Here we refer to ‘tissue’ as the compartment comprising lamina propria (LP) and LAs; we compare this with the secondary lymphoid organs: MLN, CP and OLS.

**Extended Data Fig. 2:**
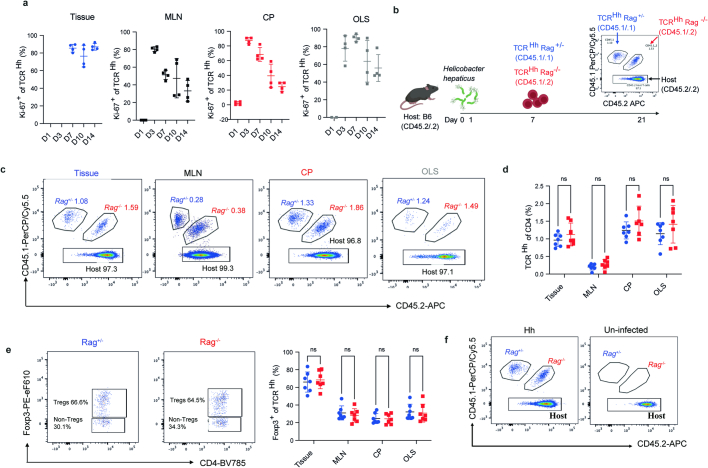
TCR^Hh^ on a Rag1^−/−^ background validates specificity of TCR^Hh^ to *Hh*. a. Frequency of Ki-67+ of TCR^Hh^ in the tissue (left), MLN (centre left), CP (centre right) and OLS (right) from day 1 to day 14 post cell transfer. b. Co-transfer of naive TCR^Hh^Rag1^+/−^ and TCR^Hh^Rag1^−/−^ into *Hh*-colonised hosts. c. Representative FACS plots of TCR^Hh^Rag1^+/−^ and TCR^Hh^Rag1^−/−^ at the indicated sites. d. Frequency of TCR^Hh^Rag1^+/−^ and TCR^Hh^Rag1^−/−^ of total CD4 T cells at indicated sites. e. Representative FACS plots (left) and frequency (right) of TCR^Hh^Rag1^+/−^ and TCR^Hh^Rag1^−/−^ Tregs of TCR^Hh^ CD4 T cells. f. Representative FACS plots of TCR^Hh^Rag1^+/−^ and TCR^Hh^Rag1^−/−^ in *Hh*-colonised and un-colonised hosts. a: 4 individual mice representative of 2 independent experiments. c-f: 7 individual mice representative of 2 independent experiments. d-e: two-way ANOVA using a Šidák’s multiple comparisons test with individual variances computed for each comparison. Source Data

Naive TCR^*Hh*^ T cells transferred into *Hh*-colonized hosts (Fig. 1b and Extended Data Fig. 1d) traffic to the secondary lymphoid organs within 24 h and onwards to the tissue approximately 1 week later (Fig. 1c,d). To pinpoint where important cellular interactions and activation steps take place, we quantified proliferation, T_reg_ cell differentiation, TCR signalling and IL-10 production across compartments. Despite exhibiting early activation in the lymphoid compartments (Extended Data Fig. 2a), at 14 days after transfer, TCR^*Hh*^ T cells in the tissue are more proliferative than their lymphoid counterparts (Fig. 1e). A larger proportion of TCR^*Hh*^ T cells differentiated into FOXP3^+^ T_reg_ cells (Fig. 1f) in the tissue than in the secondary lymphoid organs. TCR^*Hh*^*Rag1*^−/−^ control donors and uninfected recipients confirmed the specificity of TCR^*Hh*^ T cells and the dependence on cognate antigen for survival (Extended Data Fig. 2b–f).

**Extended Data Fig. 3:**
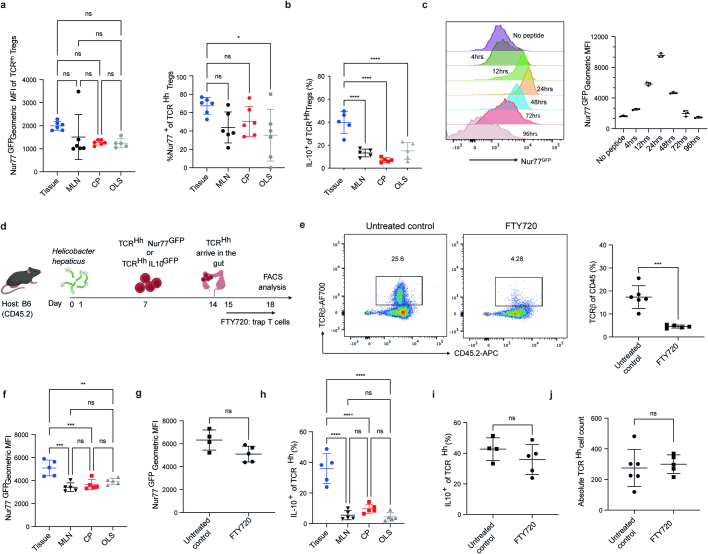
FTY720 treatment supports tissue as a dominant site of TCR signalling and IL-10 production. a. Geometric MFI (left) and frequency (right) of Nur77^GFP^ positive of TCR^Hh^ Tregs at the indicated sites. b. Frequency of IL-10+ of TCR^Hh^ Tregs at the indicated sites. c. Histogram overlay (left) and quantification of geometric MFI (right) of TCR^Hh^Nur77^GFP^ co-cultured with *Hh*-pulsed BMDCs at the indicated time points. d. Transfer of TCR^Hh^Nur77^GFP^ into FTY720-treated host mice. e. Representative FACS plots (left) and quantification (right) of TCRβ of total CD45 in peripheral blood. f. Geometric MFI of total TCR^Hh^Nur77^GFP^ in FTY720 treated hosts at the indicated sites. g. Geometric MFI of total TCR^Hh^Nur77^GFP^ in the tissue of control and FTY720 treated hosts. h. Frequency of IL-10^GFP^ positive among total TCR^Hh^ in FTY720 treated hosts. i. Frequency of IL-10^GFP^ positive among total TCR^Hh^ in the tissue of control and FTY720 treated hosts. j. Absolute TCR^Hh^ cell numbers in tissue of control and FTY720-treated hosts. a: 6 individual mice representative of 2 independent experiments. b: 5 individual mice representative of 2 independent experiments. c: 3 technical replicates representative of 2 independent experiments. e-j: 4 WT and 5 FTY720 treated mice representative of 2 independent experiments. a-b, f, h: one-way ANOVA using a Tukey’s multiple comparisons test. e, g, i-j: two-tailed t test. Source Data

As T_reg_ cell suppressor function has been shown to depend on TCRs^18^, we explored whether ongoing TCR stimulation occurs in our defined microenvironments. We crossed TCR^*Hh*^ mice to Nur77^GFP^ mice^19^ (TCR^*Hh*^Nur77^GFP^) and transferred T cells from these mice into *Hh*-colonized hosts; the proportion of cells expressing GFP and the GFP geometric mean fluorescence intensity (MFI) in the total transferred population—measures of TCR signalling—were highest in the tissue at 11 days after the transfer (Fig. 1g). Transfer of T cells from TCR^*Hh*^ mice crossed to C57BL/6 *Foxp3*^*CD2*^*Il10*^*GFP*^ reporter mice^20^ (TCR^*Hh*^*Il10*^*GFP*^) into *Hh*-colonized hosts also revealed the tissue as the site with the highest proportion of *Il10*^+^TCR^*Hh*^ T cells at day 11 after transfer (Fig. 1h). We next focused on T_reg_ cells, finding similar TCR signalling in TCR^*Hh*^Nur77^GFP^ T_reg_ cells across tissue and secondary lymphoid organs and the highest frequency of TCR^*Hh*^*Il10*^*GFP*^ cells among T_reg_ cells in the tissue (Extended Data Fig. 3a,b).

**Extended Data Fig. 4:**
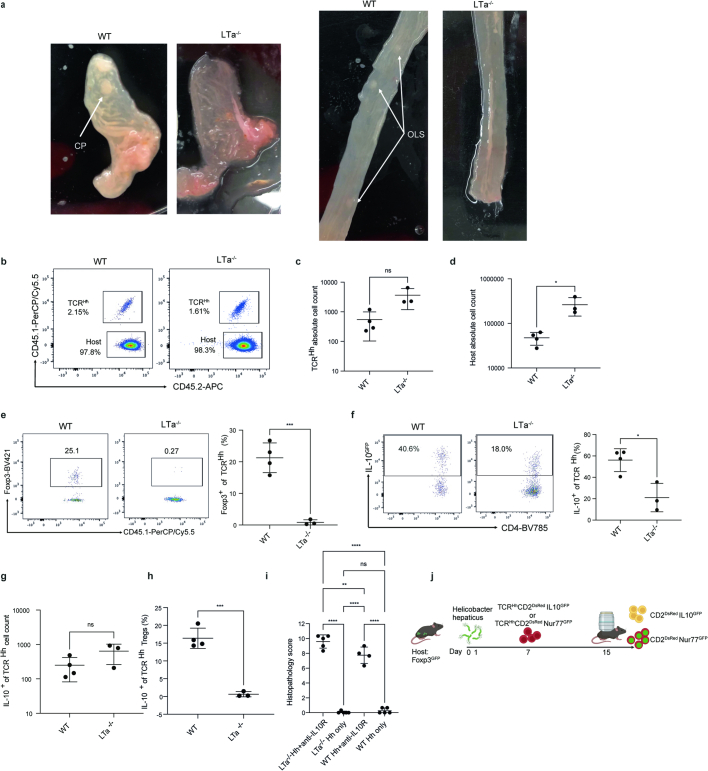
IL-10 is critical for maintaining homeostasis but LNs are not required for the pathogenic response to *Hh* in LTa^−/−^ mice. a. Representative images of the caecal patch (left) and OLS (right) in *Hh*-colonised WT and LTa^−/−^ hosts. b. Representative FACS plots of TCR^Hh^ in tissue at 10 days post naïve TCR^Hh^ cell transfer. c. Absolute TCR^Hh^ cell count in tissue of *Hh*-colonised WT and LTa^−/−^ hosts. d. Absolute host CD4 + T cell count in tissue in *Hh-*colonised WT and LTa^−/−^ hosts. e. Representative FACS plots (left) and frequency of Tregs among TCR^Hh^ (right) in tissue of WT and LTa^−/−^ hosts at 10 days post naïve TCR^Hh^ transfer. f. Representative FACS plots (left) and frequency of IL-10+ among TCR^Hh^ (right) in tissue of WT and LTa^−/−^ hosts at 10 days post naïve TCR^Hh^ transfer. g. Absolute cell count of IL-10+ among TCR^Hh^ in tissue of WT and LTa^−/−^ hosts at 10 days post naïve TCR^Hh^ transfer. h. Frequency of IL-10+ of TCR^Hh^ Tregs in tissue of *Hh*-colonised WT and LTa^−/−^ hosts at 10 days post naïve TCR^Hh^ transfer. i. Colon histopathology score of *Hh-*colonised WT and LTa^−/−^ mice with and without anti-IL-10R treatment. j. Two photon in vivo live imaging of transferred naive TCR^Hh^CD2^dsRed^Nur77^GFP^ or TCR^Hh^CD2^dsRed^IL-10^GFP^ into *Hh*-colonised Foxp3^GFP^ hosts. The caecum LA and LP were imaged. a: Representative images from 3 individual mice across 2 independent experiments. b-h: 4 WT and 4 LTa^−/−^ individual mice representative of 2 independent experiments. i: 5 WT and 5 LTa^−/−^ individual mice representative of 2 independent experiments. c-h: two-tailed t test. i: one-way ANOVA using a Tukey’s multiple comparisons test. Source Data

To determine whether TCR engagement and IL-10 production were a result of local stimulation, we used the sphingosine-1-phosphate receptor agonist FTY720 to block lymph node egress following TCR^*Hh*^ recruitment into the gut to ensure detection of local GFP induction (Extended Data Fig. 3c–e). Again, TCR signalling and the highest IL-10 production were detected in tissue TCR^*Hh*^ T cells, similar to results in untreated controls (Extended Data Fig. 3f–i). Absolute numbers of tissue TCR^*Hh*^ T cells were similar between FTY720-treated mice and untreated controls, suggesting local activation and proliferation of TCR^*Hh*^ T cells (Extended Data Fig. 3j). These data point to the tissue as a site of sustained T_reg_ cell activation and enhanced effector function in the maintenance of tolerance to the *Hh*. To test the role of the tissue LP microniche in isolation, we used mice deficient in lymphotoxin-α (*Lta*^*−/−*^ mice), which are devoid of MLNs, CP, OLS and LA^17,21^ (Extended Data Fig. 4a and data not shown). *Lta*^*−/−*^ hosts supported TCR^*Hh*^ T cell homing to tissue (Extended Data Fig. 4b–d), but in the absence of lymphoid structures, TCR^*Hh*^ T cells did not differentiate into T_reg_ cells (Extended Data Fig. 4e), indicating a non-redundant role for secondary lymphoid organs in T_reg_ cell differentiation. Similar absolute numbers of TCR^*Hh*^ T cells in *Lta*^*−/−*^ hosts retained the ability to produce IL-10 compared with controls, albeit at a lower frequency (Extended Data Fig. 4f–h). To determine whether the IL-10 produced in this reductionist system is critical for homeostasis, we blocked IL-10 signalling using a blocking IL-10 receptor (IL10R) monoclonal antibody, which resulted in inflammation (Extended Data Fig. 4i). This shows that IL-10 has a critical role in establishing homeostasis in the LP of *Lta*^*−/−*^ mice and that lymph nodes are not required for the pathogenic response to *Hh*.

Our results demonstrate that tissue interactions have a previously unrecognized role in TCR stimulation and IL-10 production by T cells that are reactive to microorganisms, including T_reg_ cells. Next, we aimed to determine whether the LP and LA niches had distinct roles in shaping the TCR^*Hh*^ response. We bred TCR^*Hh*^Nur77^GFP^ and TCR^*Hh*^*Il10*^*GFP*^ mice with *CD2*^DsRed^ mice^22^, which label T cells (resulting in TCR^*Hh*^*CD2*^DsRed^Nur77^GFP^ or TCR^*Hh*^*CD2*^DsRed^*Il10*^*GFP*^ mice) and separately transferred naive T cells from these reporter lines into *Hh*-colonized *Foxp3*^*GFP*^ hosts^23^ for in vivo two-photon live imaging (Extended Data Fig. 4j). Host LAs appear as green clusters owing to the presence of host T_reg_ cells. Transferred TCR^*Hh*^ T cells are labelled with DsRed, but co-express GFP upon TCR stimulation or IL-10 production. Three-dimensional imaging enables the visualization of green and red expression from overlapping cells (Supplementary Video 1).

Within tissue, TCR^*Hh*^Nur77^GFP+^ T cells were present within LAs (Fig. 1i). TCR^*Hh*^Nur77^GFP+^ cells were also located within the LP, spatially distant from LAs (Fig. 1i). This even distribution of recently activated T cells (Fig. 1j) suggests that TCR–major histocompatibility complex class II (MHCII) interactions occur between TCR^*Hh*^ T cells and LP-resident antigen-presenting cells (APCs), and/or rapid migration of TCR-stimulated TCR^*Hh*^ T cells out of LAs and into LP after TCR engagement. Indeed, time-lapse videos reveal that TCR^*Hh*^Nur77^GFP+^ T cells are highly motile (Supplementary Video 2). We next examined which tissue niche supported the highest production of IL-10^24^. In vivo live imaging of donor TCR^*Hh*^*CD2*^DsRed^*Il10*^*GFP*^ T cells in *Hh-*colonized *Foxp3*^*GFP*^ hosts indicated that IL-10 production by TCR^*Hh*^ cells was largely restricted to the LP (Fig. 1i,j). TCR^*Hh*^*Il10*^+^ T cells were actively motile throughout the tissue (Supplementary Video 3), suggesting a far-reaching suppressor function in response to *Hh*.

## Spatially and phenotypically distinct T_reg_ cells

**Fig. 2:**
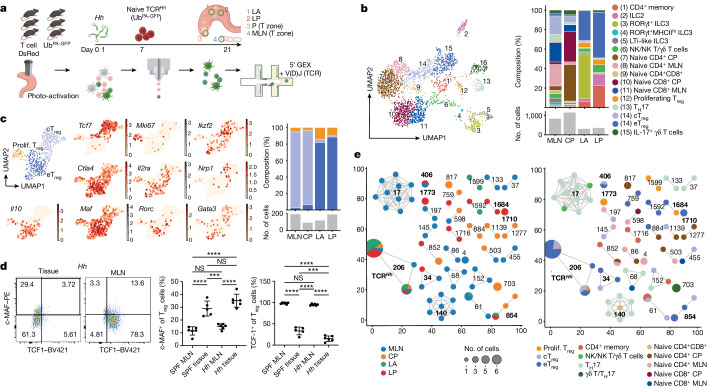
cT_reg_ cell and eT_reg_ cell populations are transcriptionally and spatially distinct. **a**, Schematic describing transfer of TCR^*Hh*^*CD2*^DsRed^Ub^PA-GFP^ T cells into six *CD2*^DsRed^Ub^PA-GFP^ hosts with photo-activation, cell sorting and scRNA-seq. DC, dendritic cell; lymph, lymphatic; Mac, macrophage; Mono, monocyte. **b**, Left, UMAP visualization of T and ILC subsets across all locations. Right, distribution of lymphoid subsets (top) and cell numbers (bottom). NK, natural killer. **c**, UMAP visualization of T_reg_ cell subsets across all locations (top left) and overlay on the UMAP plot of expression data for selected genes (middle; colours show relative expression). Right, distribution of T_reg_ cell subsets (top) and cell numbers (bottom). **d**, Left, representative FACS plots of c-MAF versus TCF1 for T_reg_ cells in tissue and MLN of *Hh*-colonized mice. Frequency of c-MAF^+^ (centre) and TCF1^+^ (right) T_reg_ cells in tissue and MLN of SPF and *Hh*-colonized mice. **e**, Clonotype network analysis of TCR^*Hh*^ T cells and host clones by location (left) and cell phenotype (right). Each fully connected subnetwork represents a ‘clonotype cluster’ and each dot represents cells with identical receptor configurations. Clonotypes with fewer than two cells were filtered out for visualization. Prolif., proliferating. **a**–**c**,**e**, One sequencing run from six combined mice. **d**, Five SPF and six *Hh*-colonized individual mice, representative of two independent experiments. **d**, One-way ANOVA using a Tukey’s multiple comparisons test. Source Data

**Extended Data Fig. 5:**
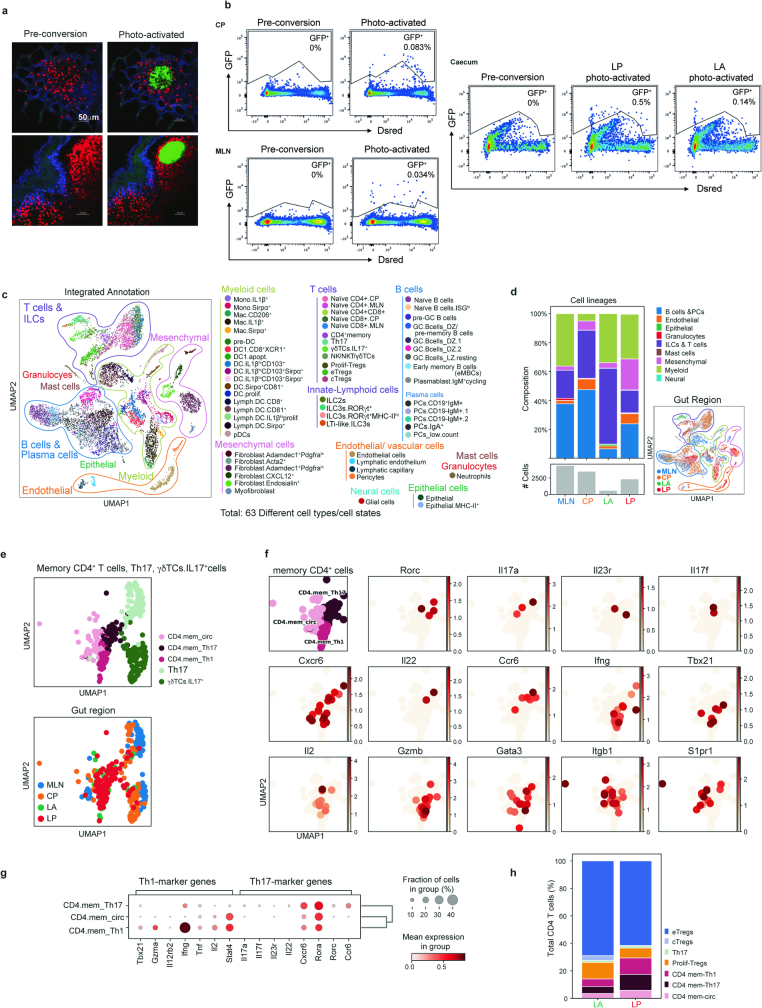
FACS cell sorting of photoactivated cells and tissue memory heterogeneity. a. Representative images of pre- and post-photoactivation of LA (top) and T cell zone of CP (bottom). b. Gating strategy of cell sorting by FACS of GFP+ cells in the caecal patch (top left), MLN (bottom left) and caecum LA and LP (right). GFP gate set on un-photoactivated samples. c. UMAP visualisation of total cell subsets (left) with detailed cell type annotations (right). d. Bar chart showing total cell subsets across all tissue niches (top left) and cell numbers (bottom left). UMAP visualisation of cell lineages by gut region (bottom right). e. UMAP of CD4 memory, Th17, and gd T cells subsets (top). Overlap of cell location (bottom). f. Gene overlays of CD4 memory subsets from all tissues. g. Gene expression of canonical Th1 and Th17 genes expressed by the CD4 memory subsets. h. Frequency bar chart showing the subsets of CD4 T cells in the LA and LP. a-h: Representative of 6 combined mice.

Because TCR^*Hh*^ T cells that have recently experienced TCR stimulation are distributed throughout the LP and LA, whereas production of the T_reg_ cell effector molecule IL-10 is highest in the LP, we examined the cellular interactions and/or molecular cues in the LP that drive TCR^*Hh*^ activation and T_reg_ cell effector functions. We used two-photon photo-activation labelling of cells in T cell niches followed by single-cell RNA sequencing (NICHE-seq) to uncover cellular composition and transcriptional states of tissue microniches^6,7^. Naive T cells from TCR^*Hh*^*CD2*^DsRed^ mice crossed to mice ubiquitously expressing photo-activatable GFP^6^ (TCR^*Hh*^*CD2*^DsRed^Ub^PA-GFP^) were transferred to *Hh-*colonized *CD2*^DsRed^Ub^PA-GFP^ hosts so that both host and donor cells were photo-activatable. DsRed was used to visualize donor cells and to mark T cell zones in host lymphoid tissue. We used two-photon microscopy to convert donor and host cells with photo-activated GFP from four regions: the T cell zones of MLNs and CP, LA and LP (Fig. 2a and Extended Data Fig. 5a,b). For each individual mouse, we photo-activated 3–10 regions per tissue location, dissociated tissues, and pooled each tissue location for sorting and sequencing. Single-cell RNA sequencing (scRNA-seq) of GFP^+^ cells using 10X, 5′ GEX and V(D)J TCR sequencing (Supplementary Data 1). A model built on previous work^15,25–31^ supported annotation of a spectrum of myeloid cells, T cells, innate lymphoid cells (ILCs), B cells, plasma cells, epithelial cells, mesenchymal cells, endothelial cells and granulocytes across all four sites (Extended Data Fig. 5c,d, Supplementary Table 1 and Supplementary Data 2–4). The relative distributions of these major intestinal cell types differ according to region, with a sizeable T cell population present across microniches (Extended Data Fig. 5d). Early memory B cells (eMBCs) were present in the LA and absent in the LP, enabling this population to be used to benchmark further LA analyses (Supplementary Data 4).

The T cell and ILC compartment comprises 16 transcriptionally distinct subsets (Fig. 2b). As expected, naive CD4^+^ and CD8^+^ T cells were mainly located in the MLNs and CP. Because photo-activation was successfully targeted to the T cell zone of each tissue, we did not detect T follicular helper cells in any compartment. As described in recent work, MHCII^hi^ type 3 ILCs (ILC3s) were identified in the MLN^12–14^, with a smaller population of MHCII^mid/low^ ILC3s in the LA. Spatial compartmentalization of ILCs within the tissue showed that ILC3s were dominant in the LA and ILC2s were dominant in the LP (Fig. 2b and Supplementary Data 3b). The LP also contained a sizeable population of memory CD4 T cells that were distinct from the γδ T cells and T helper 17 (T_H_17) cells found in the MLN (Fig. 2b and Extended Data Fig. 5e). This heterogenous population contained cells that could be broadly classified as T_H_17, T helper 1 (T_H_1) and circulating memory populations (Extended Data Fig. 5f,g). T_reg_ cells were enriched in both LP and LA at higher levels than other CD4 cell subsets (Fig. 2b and Extended Data Fig. 5h).

**Extended Data Fig. 6:**
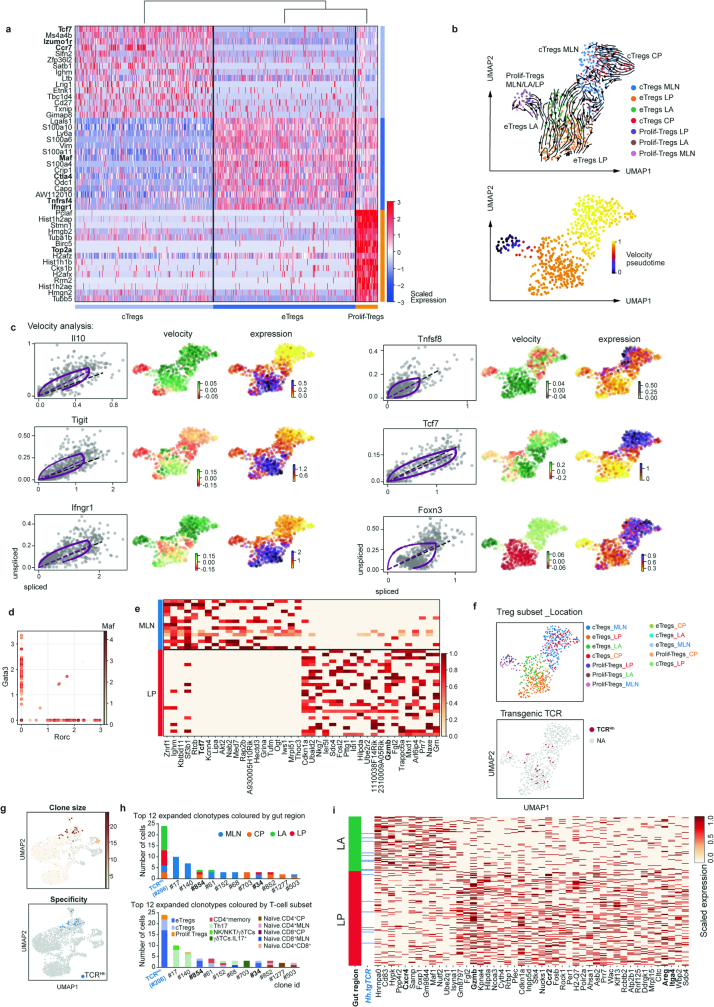
Developmental relationships between transcriptionally distinct cTregs, eTregs and proliferating Tregs. a. Heatmap of differentially expressed genes in cTreg, eTreg and proliferating Tregs. b. UMAP of Treg subsets per location, filtering out the subsets with less than 10 cells in a given location (i.e. excluding eTregs_MLN, eTregs_CP, cTregs_LA, cTregs_LP and Prolif-Tregs_CP). Arrows depict summarised scVelo differentiation trajectories (top). Heat map overlay on the Treg UMAP indicating velocity pseudotime, as calculated by scVelo (bottom). c. Velocity analysis of genes that vary across the differentiation trajectory of all Treg subsets. d. *Rorc* and *Gata3* expression of Tregs with *Maf* expression overlaid in red. e. Heatmap of differentially expressed genes between eTregs in MLN and LP. f. UMAP of the total Treg population with distributions of Treg subsets across locations (top) and overlay of TCR^Hh^ Tregs (below). g. UMAP overlay of clone size (top) and clone specificity (bottom) of TCR^Hh^ within total T cell populations. h. Barchart showing the distribution of most expanded TCR^Hh^ and host clones by location (top) and T cell subset (bottom). i. Heatmap of differentially expressed genes of host LP vs LA eTregs. Blue lines indicate gene expression profile of TCR^Hh^ eTregs in LP and LA. a-i: Representative of 6 combined mice.

Subclustering of T_reg_ cells revealed three subpopulations that were differentially distributed between secondary lymphoid organs and tissue (Fig. 2c). To highlight their distinct functional phenotype and spatial segregation, we refer to lymphoid-associated T_reg_ cells as central T_reg_ (cT_reg_) cells and tissue resident T_reg_ cells as effector eT_reg_ cells. cT_reg_ cells express *Tcf7* and *Ccr7*^32,33^, tissue eT_reg_ cells express the effector-associated molecules *Ctla4*, *Maf*, *Tnfrsf4* and *Il10*, and proliferating T_reg_ cells express histone genes (Fig. 2c and Extended Data Fig. 6a). Psuedotime analysis suggests a developmental relationship between these populations^3,15^ (Extended Data Fig. 6b,c). We sought to understand how the cT_reg_–eT_reg_ dichotomy compared with previously described intestinal T_reg_ cell subsets. Our data reveal *Rorc*, *Gata3*, *Ikzf2* and *Nrp1* expression across both cT_reg_ cell and eT_reg_ cell subsets (Fig. 2c). Although *Nrp1* expression was higher in cT_reg_ cells, it was not widely or exclusively expressed, suggesting that it would not qualify as a lineage defining marker. Expression of *Gata3* and *Rorc* was almost always mutually exclusive in T_reg_ cells (Extended Data Fig. 6d). Overlay of *Maf* expression suggests that both *Gata3* and *Rorc* T_reg_ cells are capable of upregulating the *Maf*-associated eT_reg_ cell suppressor program (Extended Data Fig. 6d). Comparison of *Rorc* T_reg_ cells in the MLN and LP again demonstrates differentially expressed *Tcf7* (Extended Data Fig. 6e), supporting the idea that *Rorc*-expressing T_reg_ cells in the MLN adopt a cT_reg_ cell phenotype. Because unbiased clustering of the T_reg_ cells separates cells on the basis of the cT_reg_–eT_reg_ divide, this suggests that these developmental states represent overriding environmental imprinting based on interactions within the microniches that are dominant over the previously described ontological distinctions. The cT_reg_ versus eT_reg_ phenotype is also observed in specific pathogen free (SPF) mice on the protein level, suggesting that these distinct T_reg_ cell phenotypes are not limited to the *Hh* setting (Fig. 2d).

To determine whether host and transferred cells were comparable for downstream analysis, we performed a T cell clonotype analysis. We detected TCR^*Hh*^ T cells (clonotype 206), which demonstrated that these cells become eT_reg_ cells in the tissue and also represent the most expanded clone in any tissue (Fig. 2e and Extended Data Fig. 6g–h). TCR^*Hh*^ T cells could be found across all locations but were enriched in LA and LP (Fig. 2e and Extended Data Fig. 6h). Uniform manifold approximation and projection (UMAP) overlay of TCR^*Hh*^ T cells onto the total T cell (Extended Data Fig. 6g) or T_reg_ cell (Extended Data Fig. 6f) pool confirmed TCR^*Hh*^ T cell distribution amongst the host clonotypes, not as a separate, unique cluster, supporting the idea that these cells can be viewed as representative of intestinal T_reg_ cells. By focusing on clones that are present in the LP, we identified expanded host clones—for example, clone 1710 that also differentiates into eT_reg_ cells within this microniche, and clone 1773 that represents proliferating T_reg_ cells within the LP (Fig. 2e). We also found evidence of shared clonotypes within (for example, clones 17 and 703) and across tissues (for example, clone 854) in different differentiation stages. Clonotypes 34 and 406 support the prediction from the trajectory analysis (Extended Data Fig. 6b) that a clonotype present as a cT_reg_ cell in a secondary lymphoid structure could further differentiate into an expanded eT_reg_ cell population within the LA and LP. Analysis of differentially expressed genes within the eT_reg_ cell populations across tissue microniches identified upregulation of *Gzmb*, *Areg*, *Ccr2* and *Itga4* in the LP and *Cxcr4* in the LA^34,35^ (Extended Data Fig. 6i). This highlights the LP as a key site favouring optimal eT_reg_ cell phenotype. Overlaying TCR^*Hh*^ T cells onto the heat map, we did not observe any phenotypic difference between the TCR^*Hh*^ T cells and their neighbouring cells within the microniches (Extended Data Fig. 6i). UMAP overlay onto the total T_reg_ cell pool confirmed that TCR^*Hh*^ was located primarily within the LP eT_reg_ cell cluster (Extended Data Fig. 6f). Owing to the small numbers of TCR^*Hh*^ T cells captured (Extended Data Fig. 6h), subsequent analysis combined host and TCR^*Hh*^ cells, as they are transcriptionally similar (Extended Data Fig. 6i). This further supports TCR^*Hh*^ as a representative, sentinel population within the T cell microniches.

**Extended Data Fig. 7:**
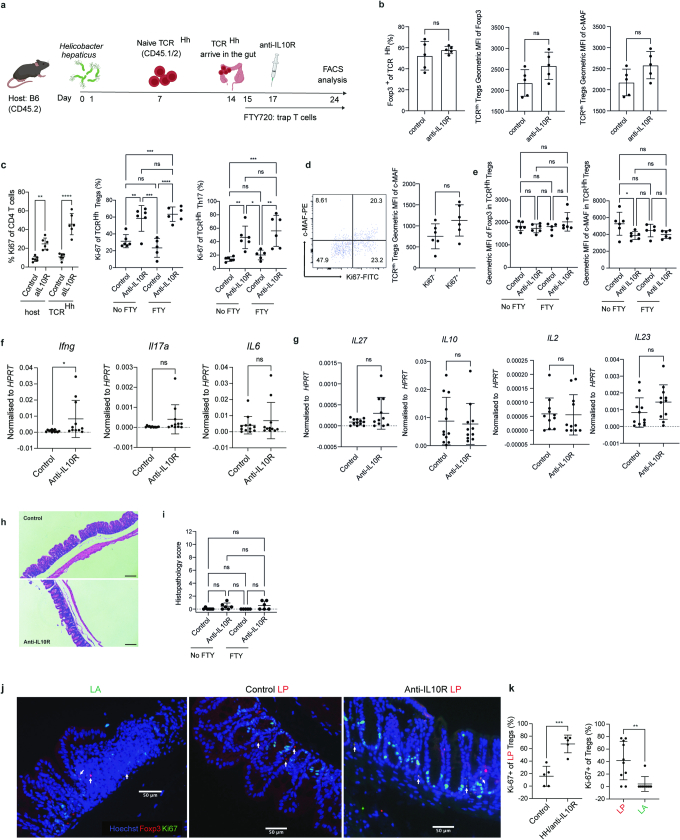
Treatment with FTY720 does not affect the phenotype and proliferation of TCR^Hh^ blocked with anti-IL-10R. a. TCR^Hh^ were transferred into *Hh* colonised host mice, treated with daily FTY720 from day 15 and anti-IL-10R at day 17. b. Frequency of Foxp3 among total TCR^Hh^ CD4 T cells (left), geometric MFI of Foxp3 expression in TCR^Hh^ Tregs (centre) and geometric MFI of c-MAF expression in TCR^Hh^ Tregs (right) in control and anti-IL-10R treated mice at day 20. c. Ki-67 of total CD4 T cells of TCR^Hh^ and host cells (left), Ki-67 of TCR^Hh^ Tregs (centre) and Ki-67 of TCR^Hh^ Th17 cells (right) in control and anti-IL-10R treated mice at day 24 with and without FTY720 blockade. d. Representative FACS plot of c-MAF versus Ki-67 in TCR^Hh^ Tregs (left) and c-MAF geometric MFI of Ki-67+ vs Ki-67- TCR^Hh^ Tregs (right). e. Geometric MFI of Foxp3 (left) and c-MAF (right) of TCR^Hh^ Tregs in control and anti-IL-10R treated mice with and without FTY720 blockade. g. Expression of *Ifnγ* (left), *Il17α* (centre) and *Il6* (right) in caecum tissue by qPCR in control and anti-IL-10R treated mice at day 24. g. Expression of *Il27* (left)*, Il10* (centre left)*, Il2* (centre right) and *Il23a* (right) in caecum tissue by qPCR in control and anti-IL-10R treated mice at day 24. h. Representative H and E staining (left top and bottom) and histopathology score (right top) of caecum tissue from control and anti-IL-10R treated mice at day 24. Scale bar represents 1 mm. i. Colon histopathology score in control and anti-IL-10R treated mice with and without FTY720 blockade. j. Representative immunofluorescent staining of Foxp3 and Ki-67 in LA (left, arrows mark Ki-67 negative Tregs), control LP (centre, arrows mark Ki-67 negative Tregs), and anti-IL-10R treated LP (right, arrows mark Ki-67 positive Tregs). k. Frequency of Ki-67 positive Tregs in the LP of control and anti-IL-10R treated mice (left) and frequency of Ki-67 positive Tregs in the LP and LA in both control and anti-IL-10R treated mice (right). b-i: 6 individual mice per group representative of 2 independent experiments. f: 11 control and 11 anti-IL-10R treated mice combined over 2 independent experiments. J-k: 5 individual mice per group representative of 2 independent experiments. b, d, f, g, k: two-tailed t test. c, e, i: one-way ANOVA using a Tukey’s multiple comparisons test. Source Data

After identifying spatially segregated cT_reg_ and eT_reg_ cells and enhanced eT_reg_ suppressor function within the LP, we blocked IL-10, a key eT_reg_ cell effector molecule, to determine the stability of the eT_reg_ phenotype in the tissue. This approach is different to the standard *Hh*/anti-IL10R colitis model^3^ because in our model, the tissue responses to *Hh* are established before we perturb IL-10 signalling. We transferred TCR^*Hh*^ T cells into colonized hosts as before, allowing TCR^*Hh*^ T cells to migrate to the gut 7 days after transfer and adopt an IL-10^+^ T_reg_ fate. We treated the hosts with FTY720 from day 8 to stop further migration from the secondary lymphoid organs and disrupted the IL-10 positive-feedback loop with an IL10Rα blocking antibody on day 10 after cell transfer (Extended Data Fig. 7a).

At 3 days after anti-IL10R treatment (13 days after TCR^*Hh*^ cell transfer), there was no significant difference between the two treatment groups in terms of the proportion or phenotypes of TCR^*Hh*^ T_reg_ cells (Extended Data Fig. 7b). However, 7 days after anti-IL10R treatment (17 days after TCR^*Hh*^ cell transfer), there was an increase in local proliferation of TCR^*Hh*^ and host T cells, with approximately 60% of TCR^*Hh*^ T_reg_ cells and TCR^*Hh*^ T_H_17 cells expressing Ki-67, which was not affected by FTY720, suggesting that these changes were generated locally (Extended Data Fig. 7c). Proliferating eT_reg_ cells expressed similar amounts of FOXP3 and c-MAF compared with non-proliferating cells (Extended Data Fig. 7d,e), suggesting that this population is phenotypically stable and capable of local proliferation. Although we observed some increased cytokine expression, we did not observe overt inflammation in FTY720-treated or untreated conditions (Extended Data Fig. 7f–i). This does not rule out the possibility that critical tolerance-establishment steps occur in the MLN, but ongoing input from the MLN is not required to maintain tissue homeostasis. To determine which niche in the tissue supported T_reg_ cell proliferation in this perturbed environment, we stained tissues with Ki-67, FOXP3 and Hoechst. This demonstrated T_reg_ cell proliferation in the LP (Extended Data Fig. 7j,k), again supporting the LP as the key location for eT_reg_ cell proliferation. These data suggest that although IL-10 is critical for initiation of tolerance, other eT_reg_ cell suppressor functions can constrain the local response.

## Myeloid compartmentalization

**Fig. 3:**
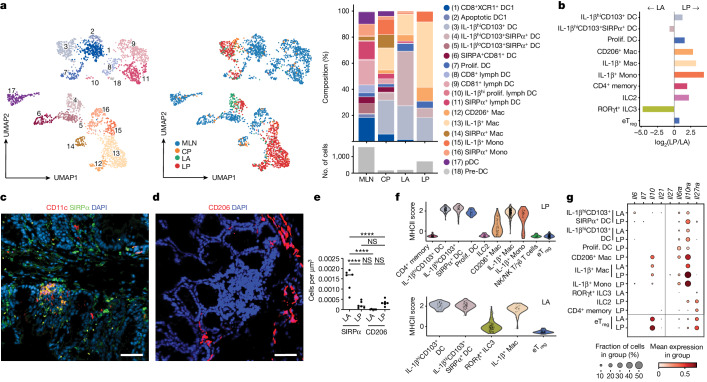
Macrophage populations are enriched in the LP microniche. **a**, Left, UMAP visualization of total myeloid subsets across all locations. Middle, UMAP of total myeloid populations by tissue microniche. Right, myeloid subset distribution (top) and total cell numbers at the indicated locations (bottom). **b**, log_2_-transformed fold difference of proportions of myeloid, T cells and ILCs in LA and LP. **c**, Representative CD11c and SIRPα immunofluorescence and DAPI staining in *Hh*-infected caecum tissue, showing an LA and surrounding LP. Scale bar, 50 μm. **d**, Representative CD206 immunofluorescence and DAPI staining in *Hh*-infected caecum tissue showing an LA and surrounding LP. Scale bars 50 μm. **e**, Densities of CD11c^+^SIRPα^+^ dendritic cells and CD206^+^ macrophages in the LA and LP microniches of *Hh-*infected caecum tissue. **f**, Violin plots of MHCII expression score across lymphoid and myeloid cells in the LP (top) and LA (bottom). Restricted to cell types with more than 30 cells per region. **g**, Relative mean expression of genes associated with STAT3 signalling and receptors in myeloid and lymphoid subsets in the LA and LP. Restricted to cell types with more than 30 cells per region. **a**,**b**,**f**,**g**, One sequencing run from four T cell compartments from six combined mice. **c**–**e**, Two independent experiments with *n* = 6 per group. Each dot represents data from one individual mouse. **e**, One-way ANOVA using Tukey’s multiple comparisons test. Source Data

The compartmentalization of enhanced T_reg_ cell function in the LP, as demonstrated by imaging and transcriptional studies, suggests that different APC populations may be stimulating and responding to eT_reg_ cells in LP and LA^36^. Mapping of monocyte/macrophage and dendritic cell subsets across the four regions showed some clustering by region; however, it did not reveal a unique APC population (Fig. 3a). We did identify an enrichment of IL-1β^hi^CD103^+^SIRPα^+^ dendritic cells within LA, although IL-1β^+^ and CD206^+^ macrophages and IL-1β^+^ monocytes were most abundant in the LP (Fig. 3b). Immunofluorescence staining of SIRPα^+^ dendritic cells shows them densely populating the LA (Fig. 3c,e), whereas CD206 staining demonstrates the presence of CD206^+^ macrophages in the LP (Fig. 3d,e). Because a composite MHCII score suggests that all myeloid populations across compartments contain MHCII^hi^ cells, antigen-driven interactions may occur between any of these groups and eT_reg_ cells (Fig. 3f).

Because IL-10 and other STAT3-activating cytokines are key to controlling homeostasis and inflammation in the intestine, we performed a search of STAT3-dependent cytokine genes across the most abundant myeloid and lymphoid subsets in the tissue. We found upregulated expression of the pro-inflammatory cytokine gene *Il6* in IL-1β^+^CD103^+^SIRPα^+^ dendritic cells resident in the LA (Fig. 3g). By contrast, the LP was dominated by *Il10*, which is expressed primarily by eT_reg_ cells, with some expression in CD206^+^ and IL-1β^+^ macrophage subsets (Fig. 3g). A small amount of *Il27* was detected in the IL-1β^+^ macrophage population, specifically in the LP. Together these data suggest potential myeloid cytokine microniches that are capable of tuning STAT3 signals, potentially establishing inflammatory (LA) and anti-inflammatory (LP) niches within the intestinal tissue. We next examined expression levels of cytokine receptor genes. Moderate levels of *Il6ra* were detected across myeloid populations, and *Il27ra* expression was largely restricted to eT_reg_ cells, CD4^+^ memory T cells and ILC2 populations. *Il10ra* was highly expressed on CD206^+^ and IL-1β^+^ macrophages and IL-1β^+^ monocytes, especially in the LP niche (Fig. 3g). Macrophage sensing of IL-10 is critical for gut homeostasis^37,38^, and our data support a potential positive-feedback loop in the LP between macrophages and eT_reg_ cells. CD206^+^ and IL-1β^+^ macrophages respond to IL-10 and produce IL-10 and IL-27^39,40^, activating STAT3 and supporting the *Maf* program in eT_reg_ cells in the LP.

## Niche disruption in colitis

**Fig. 4:**
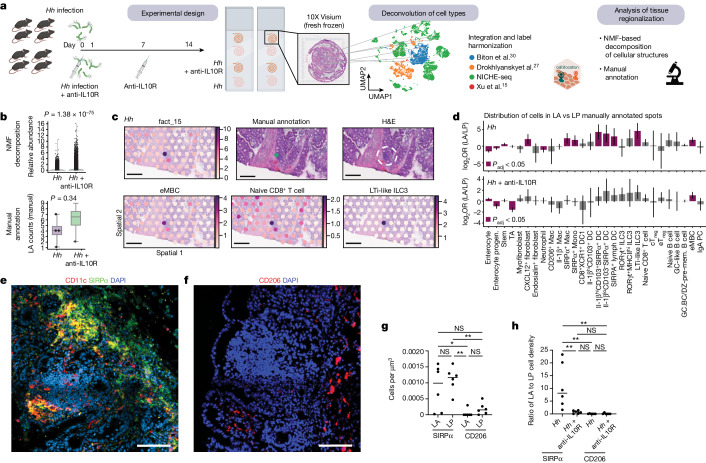
Enrichment of the LA cell signature by spatial transcriptomics analysis is diminished in inflammation. **a**, Schematic of the setup and analysis of the spatial transcriptomic experiment. **b**, Top, relative abundance of LA in *Hh*-infected mice versus *Hh*/anti-IL10R mice by NMF analysis (top) and manual annotation (bottom). **c**, H&E staining of *Hh*-infected tissue. Localization of LA-associated factor 15 by NMF decomposition as defined in Extended Data Fig. 8c (top left) and manual annotation (top centre and right) in the Visium RNA capture spots of a representative sample. Localization and normalized cell-type abundance of eMBCs (bottom left), naive CD8^+^ T cells (bottom middle) and LTi-like ILC3s (bottom right) in a representative LA. Scale bars, 200 μm. **d**, Cell-state enrichment ordered by cell lineage in the manually annotated LA versus LP spots in *Hh*-infected (top) and *Hh*/anti-IL10R (bottom) mice. Statistically significant enrichments (chi-square test, adjusted *P* value (*P*_adj_) < 0.05) are shown in magenta. Data are log_2_OR value ± s.d. progen. progenitor; TA, transit amplifying; GC, germinal centre; GC.BC/DZ-pre-mem, germinal centre B cell/dark zone and pre-memory; PC, plasma cell. **e**, CD11c and SIRPα immunofluorescence and DAPI staining in *Hh*/anti-IL10R mice, showing LA and surrounding LP. Scale bar, 100 μm. **f**, CD206 immunofluorescence and DAPI staining in *Hh*/anti-IL10R mice, showing LA and surrounding LP. Scale bar, 100 μm. **g**, Density of CD11c^+^SIRPα^+^ dendritic cells and CD206^+^ macrophages in LA and LP of *Hh*/anti-IL10R mice. **h**, Ratio of SIRPα^+^ dendritic cell and CD206 macrophage cell densities in LA vs LP in *Hh* and *Hh*/anti-IL10R mice. **a**–**d**,**i**, Four individual mice per group sequenced from two Visium slides. **e**–**h**, Two independent experiments with *n* = 6 per group. Each dot represents one mouse. **g**,**h**, One-way ANOVA using Tukey’s multiple comparisons test with single pooled variance. Source Data

**Extended Data Fig. 8:**
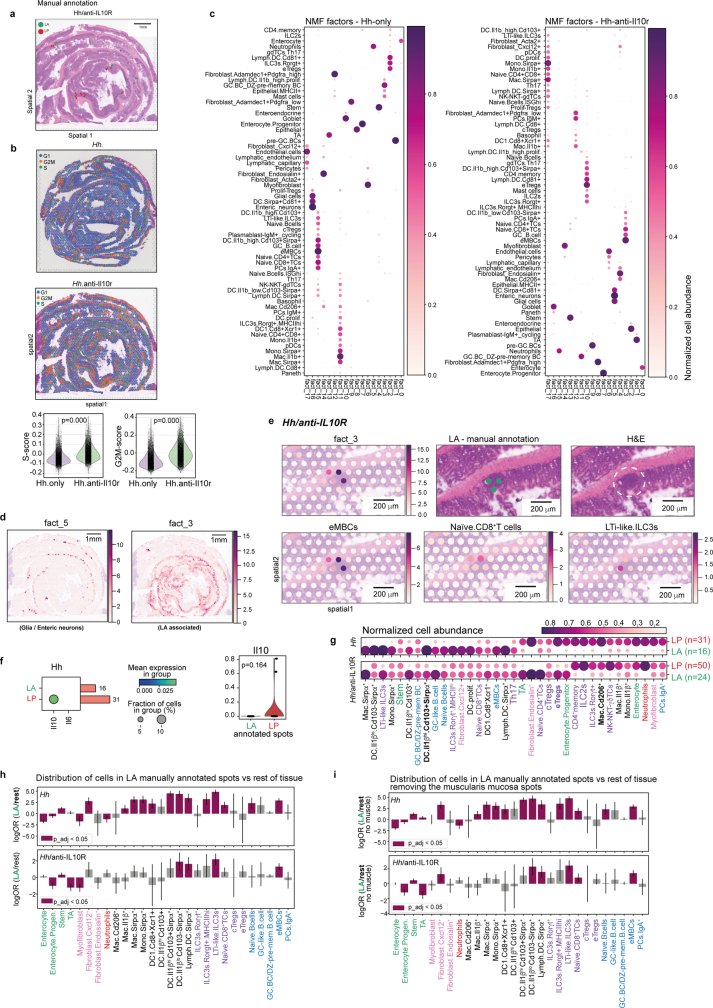
Validation of micro-niches by ST analysis and NICHE-seq. a. Representative H&E image with manually annotated LA and LP from *Hh*/anti-IL-10R. b. Visual representation of cell cycle scoring of *Hh* (top) and *Hh*/anti-IL-10R (middle) treated gut swiss-rolls with quantification of S score (bottom left) and G2M score (bottom). c. Cell type decomposition of gut swiss rolls in *Hh* (left) and *Hh*/anti-IL-10R (right) treated mice. Dotplots showing NMF weights of cell types (columns) across NMF factors (rows), which correspond to tissue microenvironments (normalized across factors per cell type by dividing by maximum values). d. Identification of microglia/enteric neurone associated factor 5 (left) and LA associated factor 3 (right). e. *Hh*/anti-IL-10R swiss roll H&E. Localisation of LA associated factor 3 by NMF -based decomposition and its overlying RNA capture spots (top left). Localisation of LA and its overlying capture spot as defined by manual annotation (top centre and right). Localisation and normalized cell type abundance of eMBCs (bottom left), naïve CD8 + T cells (bottom centre) and LTi-like ILC3s (bottom right) in representative LA. f. Dotplot of *Il10* and *Il6* gene expression in the LA and LP by ST analysis (left). Violin plot of relative distribution of *Il10* gene expression in the LA and LP by ST analysis (right). g. Cell type abundance in manually annotated LA and LP regions of *Hh* (top) and *Hh*/anti-IL-10R (bottom). Relative weights, normalized across LA/LP for every cell type. Cell type loadings represented by dot size and colour. h. Cell state enrichment (odds ratio) in the manually annotated LA vs the rest of the tissue in *Hh* (top) and *Hh*/anti-IL-10R (bottom). Statistically significant enrichments (chi-square test, p < 0.05) are shown in magenta-pink. Data show value ± SD. i. Cell state enrichment (odds ratio) in the manually annotated LA vs the rest of the tissue with muscularis mucosa removed in *Hh* (top) and *Hh*/anti-IL-10R (bottom). Statistically significant enrichments (chi-square test, p < 0.05) are shown in magenta-pink. Data show value ± SD. a-i: Representative of 4 individual mice per group (*Hh* and *Hh*/anti-IL-10R sequenced from two Visium slides.

**Extended Data Fig. 9:**
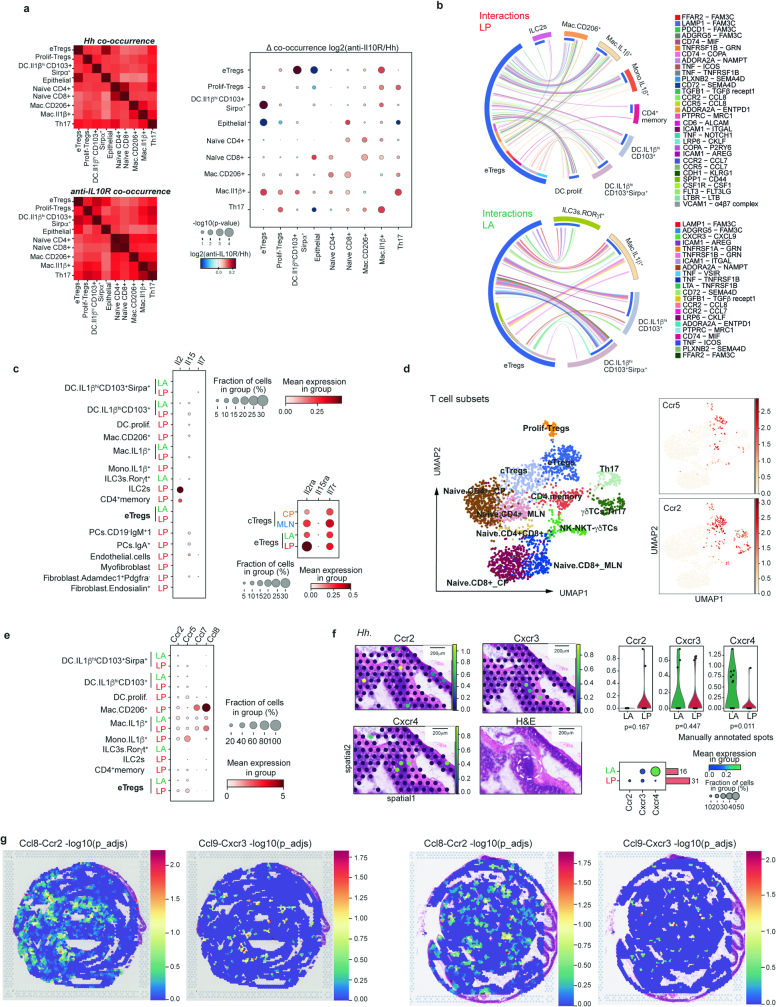
Expression of cytokines and chemokine for eTreg function. a. Co-occurrence analysis of *Hh* (left top) and *Hh*/anti-IL-10R (left bottom) with the ratio of the two conditions (right). Size of dots represents p-values and the colour represents fold change. Number of spots analysed after removing Muscularis-associated NMF factors (4405 spots) are *Hh*/anti-IL-10R: 9572 spots and *Hh*: 8815 spots. b. Chord diagrams showing significant interactions of Tregs with lymphoid and myeloid cells in LP (top) and LA (bottom). Restricted to cell types with >30 cells per region and significance p < 0.05 for interactions between any cell pair. c.Dot plot showing relative expression by NICHE-seq analysis of survival cytokine genes expressed by all the cell types with >30 cells in LA or LP regions (left) and their receptors on Treg populations (right). b. NICHE-seq analysis UMAP visualisation of T-cell subsets (left) with overlay of *Ccr5* and *Ccr2* expression (right). c. Dot plot showing expression of *Ccr2* and *Ccr5* and their ligands (*Ccl7* and *Ccl8*) in myeloid cells, T cells and ILCs isolated from the LA and LP NICHE-seq analysis. d. Localisation of *Ccr2* (top left), *Cxcr3* (top centre) and *Cxcr4* (bottom left) expression in LA and LP by ST analysis on H&E (bottom centre). Violin plots of relative distribution of *Ccr2*, *Cxcr3* and *Cxcr4* expression (top right). Dot plot of *Ccr2*, *Cxcr3* and *Cxcr4* expression in the LA and LP by ST analysis (bottom right). e. Ligand-receptor analysis on Visium data. Adjusted p-values were calculated per spot using the package stLearn and displayed in the spatial context for particular ligand-receptor pairs (‘Ccl8_Ccr2’ and ‘Cxcl9_Cxcr3’) in two representative Hh samples (above, Hh mouse 2 and below Hh mouse 4). a-c: One sequencing run from 6 combined mice. d-e: Two representative samples of 4 individual mice per group (*Hh* and *Hh*/anti-IL-10R sequenced from two Visium slides.

We aimed to extend our study to the entire intestinal tissue, validate our findings with a complementary method and include an inflammatory setting. Spatial transcriptomics is an unbiased approach to spatially map transcriptional signatures onto histological images of tissue sections^41,42^, enabling the inclusion of cells absent from the NICHE-seq pipeline. We processed frozen caecum and proximal colon tissue Swiss roll sections from mice treated with *Hh* only and anti-IL10R blockade at the time of infection to induce colitis for spatial transcriptomics using the Visium (10X Genomics) platform (Fig. 4a and Supplementary Data 5). The expression of cell cycle genes was increased across the tissue in the *Hh*/anti-IL10R setting, indicating a strong inflammatory response (Extended Data Fig. 8b). We integrated the annotations from our NICHE-seq data with data from Biton et al.^30^, Drokhlyansky et al.^27^ and Xu et al.^15^ and used the Cell2location pipeline^43^ to spatially map 102 transcriptionally distinct cell types onto the tissue sections (Fig. 4a). Manual annotation based on histology identified LAs and their overlying mRNA capture spots, distinct from the surrounding LP (Extended Data Fig. 8a). Non-negative matrix factorization (NMF) analysis identified 18 distinct transcriptional groups, covering all tissue microniches from microglia-associated (factor 5) to LA-associated (factors 3 and 15) (Extended Data Fig. 8c,d). Factor 2 includes colocalized eT_reg_ cells, ILC2s and memory CD4 T cells that were previously identified in the LP by NICHE-seq (Extended Data Fig. 8c). Manual annotation of LAs enabled validation of NMF. Both techniques show similar trends of increased LA annotated tissue microniches in the context of colitis, probably reflecting development of LA into isolated lymphoid follicles (Fig. 4b). Example overlays of factor analysis, manual annotation and haematoxylin and eosin (H&E) staining demonstrate that the LA niche-associated cell signature (eMBCs, naive CD8s and lymphoid tissue inducer (LTi)-like ILC3s) overlap (Fig. 4c and Extended Data Fig. 8e). To understand whether the LP or LA niches are key to tolerance, we examined changes in IL-10 localization during inflammation. IL-10 was enriched in the LP (Extended Data Fig. 8f), as expected on the basis of NICHE-seq data (Fig. 3g). By comparing cell-type loadings across the LA and LP microniches in the *Hh*-only setting, we could identify IL-1β^hi^CD103^+^SIRPα^+^ dendritic cells, ILC3s and eMBCs in the LA, whereas more eT_reg_ cells, CD206^+^ macrophages, ILC2s and IgA^+^ plasma cells were present in the LP, further supporting the cell enrichment in microniches identified through NICHE-seq analysis (Extended Data Fig. 8g). Expanding our analysis to the colitic *Hh*/anti-IL10R setting, we observed enrichment of some cell types, including ILC2s, in the LP; however, cells previously restricted to the LA such as IL-1β^hi^CD103^+^SIRPα^+^ dendritic cells and ILC3s could be found in the LP (Extended Data Fig. 8g). Comparing the log-transformed odds ratio (OR) of each cell type between LA and LP, we could identify cells that were significantly enriched within the LA (Fig. 4d, purple bars representing adjusted *P* value < 0.05). To include more spots in the analysis, a comparison of LA versus the rest of the tissue showed similar results (Extended Data Fig. 8h,i). As expected, this more statistically rigorous analysis showed that IL-1β^hi^CD103^+^SIRPα^+^ dendritic cells, ILC3s and eMBCs were significantly enriched in the LA of the *Hh-*only setting (Fig. 4d). The overall magnitude of enrichment quantified by log(OR) across all populations was lower during colitis, and IL-1β^hi^CD103^+^SIRPα^+^ and RORγt^+^MHC^hi^ ILC3s were no longer significantly enriched. This is consistent with cells trafficking from LA to LP, as previously observed with ILC3s^44^, and potential recruitment of additional cells into this microniche, disrupting the tolerogenic niche. Analysis of immunofluorescent staining of SIRPα^+^ dendritic cells in the *Hh*/anti-IL10R condition, showed similar distribution between the LA and LP in the context of colitis (Fig. 4e,g), which mirrors the findings from the cell-loading analysis. Similarly, staining of CD206^+^ macrophages showed no significant difference between LA and LP in colitis (Fig. 4f,g). We used analysis of the cell density from immunofluorescent staining to validate the ratio analysis in Fig. 4d. This demonstrated that the SIRPα^+^ dendritic cells, which are significantly enriched in the LA at steady state, are no longer significantly enriched in colitis (Fig. 4h). Co-occurrence analysis in *Hh* and *Hh*/anti-IL10R settings showed several immune cell populations that correlate with each other within Visium spots. By comparing the change between homeostasis and colitis, we can identify co-occurrences that significantly change in the context of inflammation. The most significantly increased co-occurrence was between CD103^+^SIRPα^+^ dendritic cells and eT_reg_ cells, again suggesting that these dendritic cells may disrupt the homeostatic interactions within tissue (Extended Data Fig. 9a).

## eT_reg_ cell–macrophage interactions in LP

eT_reg_ cells and IL-10 are more enriched in LP at homeostasis (Extended Data Fig. 8f,g), in contrast to colitis, suggesting that there are likely to be unique interactions that are lost in the context of inflammation, where dendritic cells dominate the LP. To understand which signals are important for bringing together cells of interest and contributing to local cytokine delivery, we performed CellPhoneDB analysis^45^ using NICHE-seq data from the *Hh* setting for each tissue microniche. Chord diagrams of significant interactions between any cell pair in the LP show that eT_reg_ cells are capable of interacting with all cell populations within the niche, with many potential interactions across APC subsets (Extended Data Fig. 9b). This fits with their dynamic nature, as observed previously with intravital microscopy (Supplementary Video 3). The interaction diagram for LA shows that eT_reg_ cells have more interactions with IL-1β^+^CD103^+^SIRPα^+^ dendritic cells in particular (Extended Data Fig. 9b).

**Fig. 5:**
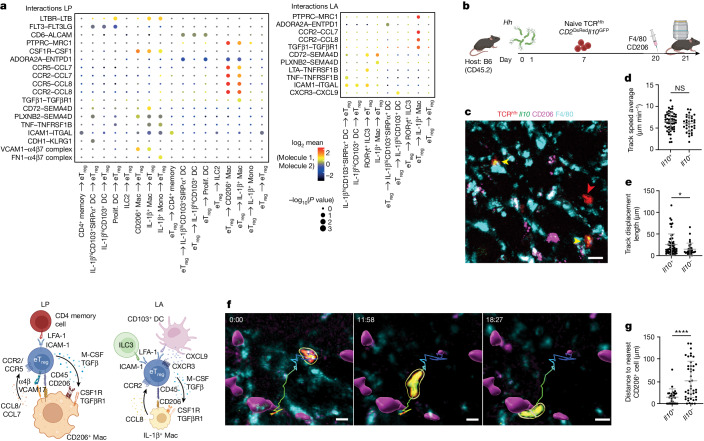
In vivo live imaging demonstrates *Il10*^+^ TCR^*Hh*^ T cells interacting with CD206^+ =^ macrophages in the LP. **a**, CellPhoneDB analysis of receptor–ligand interactions in LP (top left) and LA (top right), restricted to cell types with more than 30 cells per region and limited to unique pairs with *P* < 0.01. MHCII interactions are excluded. Schematic summarizing the most relevant cell–cell interactions (bottom). **b**, Two-photon in vivo live imaging of TCR^*Hh*^*CD2*^DsRed^*Il10*^*GFP*^ T cells transferred into *Hh*-colonized hosts labelled in vivo with CD206 and F4/80 fluorescent antibodies. **c**, Representative image of donor TCR^*Hh*^*CD2*^DsRed^*Il10*^*GFP*^ T cells in the LP. Arrowheads indicate *Il10*^−^ TCR^*Hh*^ (red) and *Il10*^+^ TCR^*Hh*^ (yellow) T cells. Scale bar, 20 μm. **d**, Average track speed of *Il10*^+^ and *Il10*^−^ TCR^*Hh*^ T cells in the LP. **e**, Track displacement length of *Il10*^+^ and *Il10*^−^ TCR^*Hh*^ T cells in the LP. **f**, Sequential video stills showing an *Il10*^+^ TCR^*Hh*^ T cell moving from one CD206^+^ cell to another in the LP. Scale bars, 10 μm. **g**, Distance from *Il10*^+^ and *Il10*^−^ TCR^*Hh*^ to the nearest CD206^+^ cell in the LP. **a**,**b**, One sequencing run from six combined mice. **d**–**h**, Representative images and combined data from six individual mice over two independent imaging experiments. **d**,**e**,**g**, Mann–Whitney test. Source Data

CellPhoneDB analyses of unique cell-pair interactions with a more stringent *P* < 0.01 in the LA and LP catalogue key predicted interactions between cells in each tissue microniche (Fig. 5a). In addition to differences in interactions with APCs, the eT_reg_ cells in the LP had the strongest ICAM1–ITGAL interactions with CD4 memory T cells and LTBR–LTB interactions with ILC2s, whereas eT_reg_ cells in the LA could interact with ILC3s through ICAM1–ITGAL (Fig. 5a). These eT_reg_ cell–ILC2 interactions observed across experimental analyses (Fig. 2b and Extended Data Fig. 8c,g) provide a potential source of IL-2 for the eT_reg_ cells within the LP niche (Extended Data Fig. 9c). Of particular interest are the chemokine–chemokine receptor and integrin pairs that bring APCs and eT_reg_ cells together. Within the LP, eT_reg_ cells have the strongest CCR2–CCL8, PTPRC–MRC1, CSF1–CSFR1 and VCAM1–A4B7 pathway interactions with CD206^+^ macrophages, suggesting that these cells may have a dominant role beyond TCR engagement (Fig. 5a). Within the LA, the CCR2–CCL8, PTPRC–MRC1 and CD72–SEMA4D interactions connect eT_reg_ cells with IL-1β^+^ macrophages, and CXCR3–CXCL9 and ICAM1–ITGAL interactions connect eT_reg_ cells with LA dendritic cell populations (Fig. 5a). On the basis of the unbiased CellPhone analysis we further analysed CCR2 expression in the T cell compartment, which is highest in eT_reg_ cells and some T_H_17 cells (Extended Data Fig. 9d,e). The ligands, CCL7 and CCL8, are most highly expressed by CD206^+^ macrophages, further supporting a potential key role for eT_reg_ cell–CD206^+^ macrophage interactions in the LP niche (Extended Data Fig. 9e). Compartmentalization of CCR2 in LP and CXCR3–CXCR4 in LA is further validated by expression levels and receptor–ligand analyses in spatial transcriptomics data (Extended Data Fig. 9f,g).

On the basis of the CCR2–CCL8 axis highlighted in the CellPhoneDB data and differential expression of CCR2 in LP eT_reg_ cells (Fig. 5a and Extended Data Figs. 6i and 9d–g), we hypothesized that eT_reg_ cells interact directly with CD206^+^ macrophages in the LP. To test this, we performed in vivo two-photon live imaging of TCR^*Hh*^*CD2*^DsRed^*Il10*^*GFP*^ T cells transferred into *Hh*-colonized hosts and labelled in vivo with F4/80 and CD206 antibodies (Fig. 5b). Imaging the LP showed *Il10*^+^ TCR^*Hh*^ T cells intimately associated with CD206^+^F4/80^+^ macrophages, in contrast to their *Il10*^−^ TCR^*Hh*^ T cell counterparts (Fig. 5c). Despite similar cell track speeds, *Il10*-GFP^+^ TCR^*Hh*^ cells exhibited greater displacement (Fig. 5d,e). This suggests that *Il10*-GFP^+^ TCR^*Hh*^ T cells are highly motile over large areas of LP, whereas *Il10*-GFP^−^ TCR^*Hh*^ T cells are motile within a more confined area. Analyses of time-lapse images show *Il10*-GFP^+^ TCR^*Hh*^ cells moving from one CD206^+^ macrophage to another (Fig. 5f and Supplementary Video 4). The result of motile eT_reg_ cells interacting with CD206^+^ macrophages in the LP is that the distance from any *Il10*-GFP^+^ TCR^*Hh*^ T cell to a CD206^+^ macrophage is significantly smaller compared with *Il10*^−^ TCR^*Hh*^ cells, with about one-third of cells being in contact with a CD206^+^ macrophage at one time (Fig. 5g).

## Discussion

In this study, we follow the natural history of TCR^*Hh*^ as a model of microorganism-reactive T_reg_ cells as they acquire and maintain immune regulatory function in the intestine. By examining cells in anatomical microniches, we have revealed the importance of key interactions to enhance eT_reg_ cell antigen stimulation and effector function. The cT_reg_–eT_reg_ differentiation trajectory identified using TCR^*Hh*^ T cells and mirrored in endogenous T_reg_ cells suggests that overriding environmental imprinting based on interactions within microenvironments drives T_reg_ phenotype, overlaying this paradigm on the *Gata3–Rorc* dichotomy. Even among eT_reg_ cells, the LP—and not the LA—is the site of enhanced eT_reg_ cell function, including the production of AREG, GZMB and IL-10.

The LP is unique in its cellular makeup, supporting tolerance at steady state. This microniche is inhabited by eT_reg_ cells and a diverse set of APCs, including CD103^+^ dendritic cells, CD206^+^ macrophages, IL-1β^+^ macrophages, and monocytes that express the highest levels of IL10R, making them prime targets for IL-10 produced by eT_reg_ cells. Once this tolerogenic niche is established, eT_reg_ cells can proliferate within this microniche to control pathology. However, the LP niche is perturbed in the context of inflammation, with several cell types including CD103^+^SIRPα^+^ dendritic cells previously confined to the LA, recruited into the LP, again pointing to this tissue as key to mucosal tolerance. Within the LP, IL-10-producing T_reg_ cells are motile and participate in serial interactions with CD206^+^ macrophages. eT_reg_ cells and CD206^+^ macrophages express several molecules capable of potentiating these interactions, including attraction through CCR2–CCL8, adhesion through VCAM1–α4β7 and CD45–CD206 and immune activation and control through TCR–MHC and IL-10–IL10R.

This work highlights the importance of the eT_reg_ cell program, and the motility and stability of eT_reg_ cells, which are uniquely poised to interact with cells across a large barrier surface. By characterizing their niche in addition to their cellular phenotype, we have uncovered several pathways that may be enhanced or disrupted to control pathology. Information gained from the study of microniches can be used for targeted interventions, cell therapy and vaccination strategies to support eT_reg_ cell recruitment, activation, differentiation, survival and function in the inflammatory niche of the intestine and other organs.

## Methods

### Mice

C57BL/6 J (B6) and *Foxp3*^*GFP*^, *Rag1*^*−/−*^, *CD2*^DsRed^, *Foxp3*^*CD2*^*Il10*^*GFP*^, Ub^PA-GFP^ and Nur77^GFP^ mice were bred and maintained under specific pathogen free conditions in an accredited animal facility at the University of Oxford. *Lta*^*−/−*^ mice were purchased from Jackson Laboratories. HH7-2tg TCR transgenic mice, referred to as TCR^*Hh*^ here, were provided by D. R. Littman. TCR^*Hh*^ mice were bred with *CD2*^DsRed^ mice and either *Foxp3*^*CD2*^*Il10*^*GFP*^ or Nur77^GFP^ mice to generate TCR^*Hh*^*CD2*^DsRed^*Il10*^*GFP*^ or TCR^*Hh*^*CD2*^DsRed^Nur77^GFP^, respectively. TCR^*Hh*^ mice were bred to *Rag1*^*−/−*^ to generate TCR^*Hh*^*Rag1*^*−/−*^ mice. UB^PA-GFP^ mice were bred to *CD2*^DsRed^ and TCR^*Hh*^ mice to generate *CD2*^DsRed^Ub^PA-GFP^ and TCR^*Hh*^*CD2*^DsRed^Ub^PA-GFP^, respectively.

Mice were free of *Helicobacter* spp. and other known intestinal pathogens, were age- and sex-matched and between 6 and 12 weeks old. Animals were randomly assigned to experimental group, and cages contained mice of all different experimental groups. All experiments were conducted in accordance with the UK Scientific Procedures Act of 1986, and by persons holding a personal license. The project licence governing the mouse studies was reviewed by the University of Oxford’s Animal Welfare and Ethical Review Board and approved by the Home Office of His Majesty’s Government.

No statistical methods were used to predetermine sample size. Sample sizes were based on previous similarly designed experiments from our research group. The spatial transcriptomics experiment included four mice per group to balance statistical power with cost. For other experiments we aimed for a minimum of five mice per experimental group. Exact mouse numbers for each experiment are included in the figure legends. Mice were assigned to different experimental groups at random. Mice were co-housed and littermate when possible. Each cage contained all treatment conditions. Animal studies were not blinded. Histopathology scoring was conducted by two independent assessors, one of whom was blinded.

### Flow cytometry

Mouse cells were stained with combinations of the following monoclonal antibodies, all purchased from Biolegend, Invitrogen, or eBioscience: CD4 (RM4-5), TCRβ (H57-957), CD45.1 (A20), CD45.2 (104), CD11c (N418), CD11b (M1/70), anti-human CD2 (TS1/8), CXCR5 (L138D7), PD-1 (J43), FOXP3 (FJK-16s), RORγt (Q31-378), Ki-67 (SolA15). Dead cells were excluded using efluor 780 fixable viability dye (eBioscience). For transcription factor staining, cells were stained with surface markers prior to fixation and permeabilization using the FOXP3 staining buffer kit (eBioscience) according to manufacturer instructions.

### Immunofluorescence staining

Swiss-rolled caecum tissues were fixed overnight at 4 °C in PLP buffer (1% paraformaldehyde, l-lysine 0.2 M pH 7.4 and 32 mg NaIO_4_). Then, tissues were dehydrated in 20% sucrose for at least 4 h at 4 °C and embedded in OCT compound (Avantor). Seven-micrometre cryosections were rehydrated, blocked and permeabilized with PBS, 1% goat serum, 1% BSA, 0.3 M glycine, 0.3% Triton X-100 for 1 h at room temperature. Sections were stained with the following antibodies: Alexa Fluor 488 anti-mouse CD172a (SIRPα) (clone P84, 5 μg ml^−1^ Biolegend), Alexa Fluor647 anti-mouse CD11c (clone N418, 5 μg ml^−1^ Biolegend) or FITC/Alexa Fluor 594 anti-mouse CD206 (clone C068C2, 5 μg ml^−1^ Biolegend) and anti-mouse CD64 (clone X54-5/7.1, 4 μg ml^−1^ Biolegend). Sections were stained overnight at 4 °C. Before imaging, nuclei were counterstained with Hoechst. Images were acquired using Zen Blue software on a ZEISS 980 Airyscan inverted microscope equipped with a motorized stage. Diode laser lines were used for excitation: violet (405 nm), blue (488 nm), yellow (514 nm) and red (639 nm). All images were acquired with a 25× (NA 0.8) LD LCI Plan-Apochromat oil-immersion objective.

Colon tissue was embedded in OCT (Tissue-Tek) as Swiss rolls and sectioned at 7 μm. Slides were fixed with 3.7% formalin (Merck) and blocked with 10% donkey serum (Sigma Aldrich) and 1% Fc block (eBioscience) in permeabilization buffer (Foxp3/Transcription factor staining buffer set, eBioscience). B220 (RA3-6B2), CD4 (RM4-5), MHC Class II (M5/114.15.2), gp38 (8.1.1), IgD (11-26 c.2a) and BCL6 (IG191E/A8) (all Biolegend) were stained overnight in blocking serum.

### Isolation of lymphocytes from spleen, lymph node and intestinal tissue

Intestinal tissues were washed twice in RPMI (Sigma Aldrich)/10%FCS/5 mM EDTA at 37 °C with agitation for 25 min to remove epithelial cells. CP and OLS were removed under 40× bright-field microscopy using a scalpel and a 16G needle and syringe, respectively. Remaining colon and caecum tissue, OLS and CP were digested for 40 min at 37 °C with agitation in RPMI/10% FCS/15 mM Hepes with 100 U ml^−1^ collagenase VIII (Sigma Aldrich) and 20 mg ml^−1^ DNase I (Sigma Aldrich). Leukocytes from colon and caecum tissue were recovered at the interface of a 40/70% Percoll gradient (Fisher Scientific).

Spleens and MLNs were mechanically disrupted, and splenic red blood cells were lysed with ACK lysis buffer.

Peripheral blood was collected by cardiac puncture and red cells were lysed with ACK lysis buffer.

### *Hh* culture and oral gavage

*Hh* NCI-Frederick isolate 1 A (strain 51449) was grown on blood agar plates containing 7.5% laked horse blood (Thermo Scientific) and Skirrow Campylobacter supplement (Oxoid) under microaerophilic conditions at 37 °C with agitation. Cultures were expanded for 48 h in Tryptone Soy Broth (TSB, Fisher) containing 10% FCS (Gibco) and the above antibiotics. The concentration of bacteria was determined by optical density (OD) analysis at 600 nm. Mice were fed 1 × 10^8^ colony-forming units of *Hh* (equivalent to 1 OD unit) by oral gavage using a curved 22G needle for a total of 2 doses 24 h apart.

### Lymph node lymphocyte egress blocking experiment

Host mice were treated every 24 h with 1 mg kg^−1^ of the sphingosine-1-phosphate antagonist, Fingolimod (FTY720, Sigma Aldrich) via intraperitoneal injection at the indicated timepoints after naive TCR^*Hh*^ cell transfer.

### Sorting and adoptive transfer of naive TCR^*Hh*^ T cells

Naive T cells were isolated from TCR^*Hh*^ mice splenocytes and sorted by flow cytometry as CD45^+^CD3^+^CD4^+^CD44^low^CD62L^hi^Vβ6^+^ (Extended Data Fig. 1d), with up to 2% contamination with nT_reg_ cells. All monoclonal antibodies were purchased from Biolegend or eBioscience: CD3 (145-2C11), CD11b (M1/70), CD11c (N418), B220 (RA3-6B2), CD62L (MEL-14), TCRVβ6 (RR4-7), CD44 (IM7), CD45 (30-F11), CD4 (RM4-5). Sorted cells (2 × 10^3^ or 5 × 10^4^) were injected by intravenous injection into the tail vein for flow cytometric or in vivo live imaging respectively.

### In vitro co-culture

Bone marrow stem cells were extracted from wild-type mouse femurs and cultured for 7 days in RPMI (Sigma) supplemented with 1% penicillin-streptomycin (Sigma), 10% FCS (Life Technologies), 1% Glutamax (Invitrogen) and 20 ng ml^−1^ mouse GM-CSF (Peprotech). Bone marrow-derived dendritic cells (BMDCs) were plated at a density of 1 × 10^4^ cells per well overnight. *Hh* peptide (1 mg ml^−1^, Genscript) was added 1 h prior to plating 1 × 10^5^ sorted naive TCR^*Hh*^Nur77^GFP^ T cells in RPMI/10% FCS/1% Glutamax/1% penicillin-streptomycin and 50 mM β-mercaptoethanol (Life Technologies). Anti-mouse I-A/I-E antibody was added at 4, 12, 24, 48, 72 and 96 h after the plating of TCR^*Hh*^Nur77^GFP^ T cells.

### Two-photon microscopy

Mice were anaesthetized with isoflurane, the caecum exposed and immobilized with a suctioning imaging window^46^. Samples were illuminated with 910 nm <70 fs pulsed light from Mai-Tai laser and collected using a 20× water-dipping lens and the spectral detector of a Zeiss 880 multiphoton microscope (Carl Zeiss). Images were linearly unmixed using the Zen software (Carl Zeiss) to separate autofluorescence, collagen, eGFP, DsRed and Texas-red dextran based on single-colour controls. All in vivo live images of the LA and LP were performed in the caecum due to ease of access.

Intravital videos were drift-corrected based on mucus or collagen signal. Images were smoothed using a Gaussian filter for display.

### Image analysis

Intravital microscopy images were analysed using Imaris 9 (Bitplane). Following unmixing, autofluorescence was subtracted from DsRed and GFP channels. GFP^+^ and DsRed^+^ cells were marked using the Surface Creation Wizard, and their co-expression and location within LP and LA compartments were recorded.

Immunofluorescence images were analysed using Imaris 10.0 (Bitplane). LAs were defined based on DAPI staining showing nuclear density and surrounding epithelial morphology, allowing for unbiased region selection. LP was chosen based on location and cellular density based on DAPI stain so that each region of interest collected and analysed for LA contained at least six LP surfaces of matching size. Surfaces for each cell type of interest were created using CD11c^+^ for BMDCs, CD206^+^ for CD206^+^ macrophages using the Surface Creation Wizard, which was applied to all images collected with the same conditions. CD11c^+^ surfaces were further subdivided based on median SIRPα staining levels within the surface.

### Photo-activation

*CD2*^DsRed^Ub^PA-GFP^ hosts were colonized with *Hh* on day 0. Seven days later 50,000 naive TCR^*Hh*^*CD2*^DsRed^Ub^PA-GFP^ cells were transferred into the *Hh-*colonized *CD2*^DsRed^Ub^PA-GFP^ hosts so both host and donor cells were photo-activatable. On day 21 the MLN and caecum were removed from 6 mice. The caecum was shaken at 37 C in RPMI + BSA + EDTA for 40 min and 20 min to remove the epithelium. The CP was separated from the rest of the tissue, and the remaining tissue was divided into one third without photo-activation as a negative control for FACS gating, one third for LA photo-activation, and one third for LP photo-activation.

Samples were maintained in RPMI + BSA+Hepes on ice in the dark for the duration of the experiment with cold media flowed over the tissue during photo-activation. A Zeiss 880 upright multiphoton microscope (Carl Zeiss, Germany) fitted with two tunable lasers (Mai-Tai tuneable BB laser 710–990 nm, pulse width <80 fs and Mai-Tai tuneable 690–1040 nm, pulse width <70 fs) and a 20× water-dipping objective was used for tissue photo-activation. The microscope was set to dynamically unmix GFP, DsRed, and collagen based on pre-collected single-colour controls. Samples were imaged with 910 nm light to identify regions of interest based on *CD2*^DsRed^. MLN and CP T cell zones were defined as the densest T cell regions without gaps to exclude B cell zones. LA regions were defined as a cluster of cells with a diameter of at least ten *CD2*^DsRed^ cells. LP region was defined as a region containing *CD2*^DsRed^ cells distal to LA. After ROI definition, the second laser was turned on at 740 nm while imaging live. GFP photo-activation was observed dynamically to ensure sufficient photo-activation without toxicity. For each mouse 3–10 regions were photo-activated for each tissue microniche. Each photo-activation region comprised approximately 40,000 µm^3^ of tissue (70 µm diameter × 10 µm depth).

After photo-activation, tissues were minced and digested for 30 min in RPMI+Hepes with 100 U ml^−1^ collagenase VIII (Sigma Aldrich) and 20 mg ml^−1^ DNase I (Sigma Aldrich). The digested tissue from the six mice was pooled per tissue microniche into one sample. Isolated cells were stained with efluor 780 fixable viability dye (eBioscience). Photo-activated GFP^+^ cells were sorted using a four laser BD FACSAria III based on gates defined by unactivated samples from the same mice (Extended Data Fig. 5b).

### Single-cell RNA library construction and sequencing

For scRNA-seq experiments, the Chromium Single Cell 5′ version 2 reagent kit and Chromium Single Cell Mouse TCR Amplification Kit (10x Genomics) were used. Sorted cells were loaded onto each channel of the Chromium Chip K following the manufacturer’s instructions and the chip was inserted in the Chromium Controller for droplet encapsulation. cDNA synthesis, amplification, gene expression (GEX) and targeted TCR was performed on single cells, according to the manufacturer’s protocol (CG000331). Sequencing was performed on the Illumina Novaseq 6000 system. Gene expression libraries were sequenced at a targeted depth of 50,000 reads per cell, using the following parameters: Read1: 28 bp i7:10 bp, i5: 10 bp, Read2: 98 bp. TCR libraries were pooled at a ratio of 1:10 with the GEX libraries and sequenced at a target depth of 5,000 reads per cell.

### scRNA-seq analysis

#### Pre-processing of 10x Genomics scRNA-seq and scTCR-seq data

scRNA-seq raw sequencing data were processed using the CellRanger “multi” software (version 6.1.1, 10x Genomics) with the mm10 2020-A mouse reference genome (official 10X mouse pre-built reference). Single-cell TCR-sequencing (scTCR-seq) data were aligned and quantified using the CellRanger ‘multi’ software (v.6.6.1) and the reference vdj_GRCm38_alts_ensembl-5.0.0 was used with default settings.

#### Quality control and processing of scRNA-seq data

Data pre-processing was performed using the ScanPy workflow (v. 1.8.1)^47^. ScanPy (v. 1.7.1), Anndata (v. 0.7.5), Pandas (v.1.2.3), NumPy (v.1.20.1), and Python (v.3) were used to pool single-cell counts and conduct downstream analysis. For each run, SoupX algorithm^48^ was run with default parameters to remove ambient mRNA from the count matrix. Doublet detection was performed using the Scrublet algorithm (https://github.com/AllonKleinLab/scrublet^49^) with percolation step, as previously described^50^. Additional doublet exclusion was performed throughout downstream processing based on unexpected co-expression of canonical markers, such as *Cd3d* (T cells) and *Cd19* (B cells). Cells with fewer than 1,000 unique molecular identifier (UMI) counts, fewer than 600 detected genes and more than 15% mitochondrial reads were excluded from downstream analysis. Genes were filtered out for expression in less than three cells. Gene expression for each cell was normalized (scanpy.pp.normalize_total, scaling factor 10,000) and log-transformed (scanpy.pp.log1p). Downstream analyses included variable gene detection (scanpy.pp.highly_variable_genes) and data feature scaling (sc.pp.scale). Cell cycle score was calculated using the expression of the cell cycle genes in Supplementary Table 1. Cell cycle score, UMI counts, the percentage of mitochondrial reads, and the percentage of Ig reads (calculated based on the genes provided in Supplementary Table 1) were regressed out during scaling the data. Dimensionality reduction (scanpy.tl.pca, based on highly variable genes) and Leiden-graph-based clustering (scanpy.tl.leiden, with clustering resolution manually adjusted, 0.3–1.5) were carried out. Cell lineages were annotated on the basimarker gene expression for each cluster (sc.tl.rank_genes_groups, method = ‘wilcoxon’).

#### Cell-type annotation with CellTypist

CellTypist is a cell-type database, server and pipeline for automatic annotation of scRNA-seq developed at Teichman lab (https://github.com/Teichlab/celltypist, https://www.celltypist.org). To assemble a mouse intestinal reference dataset, scRNA-seq data were collected from eight publications covering different cell lineages from small and large intestine as well as one additional dataset of sorted B cells from spleen, to cover in detail the annotation of germinal centre B cell populations (Supplementary Data 2).

For each dataset, the raw count matrix was downloaded along with the accompanying cell meta information. After removing trivial cell types annotated by the original studies (for example, ‘doublets’ and ‘unresolved’), a total of 171,271 cells were obtained representing all major cell populations in the mouse gut. Cell-type names from different datasets were next standardized to achieve a common scheme of nomenclature. Specifically, the similarity of transcriptomes between each pair of cell types across datasets was examined and the two cell types were merged only if they corresponded to a single cell type (for example, ‘enteroendocrine cell’ from the Tabula Muris was renamed to ‘enteroendocrine’ as was used in Biton et al.^30^). Finally, after in-depth inspection, 126 cell types were harmonized from the eight datasets. A CellTypist model then was created based on logistic regression classifiers, as described in detailed^48^. The model is publicly available at https://celltypist.cog.sanger.ac.uk/models/Mouse_Gut_Casado/v2/Adult_Mouse_Gut.pkl. Cell identities were predicted using the resulting model, followed by manual curation. Cells from each lineage were further subclustered and Leiden clustering was repeated for fine-grained annotation of the cell types and states. A description of cell-type annotations for each lineage is provided in Extended Data Fig. 5c and Supplementary Data 3 and 4. The differentially expressed genes for the cell types in each lineage can be found in the Supplementary Table 1.

For prediction on cycling regulatory T cells (prolif. T_reg_ cells), eT_reg_ and cT_reg_ cells were used as a training reference. The model was built applying default parameters, and prediction was performed without majority voting.

#### scTCR-seq downstream analysis

The Python package scirpy (v. 0.12.2)^51^ was used to extract the V(D)J sequence information from the CellRanger output file filtered_contig_annotations.csv. Productive TCRαβ chains were determined using the scirpy.tl.chain_qc function, and cells without V(D)J data or with two pairs of productive TCRαβ chains were removed from the analysis. Clonotypes were defined with the function scirpy.tl.define_clonotypes based on the CDR3 nucleotide sequence identity and the V-gene usage for any of the TCR chains (either VJ or V(D)J need to match). For cells with dual TCRα or TCRβ chains, any of the primary or secondary receptor chain matching was considered for the clonotype definition. Clonotype networks were constructed using the pp.ir_dist function to compute distances between CDR3 nucleotide sequences (using identity as a metric) and tl.define_clonotype_clusters function to designate the clonotype clusters, removing the clonotypes with less than two cells. The TCR metadata were combined with the transcriptome data for downstream analysis and comparison of different T cell populations. *Hh*-specific TCR data were retrieved from Xu et al.^15^, and TCR sequences were obtained from https://www.ncbi.nlm.nih.gov/nuccore and mapped using the IMGT/V-QUEST alignment tool^52^. Hh.7-2 transgenic TCR (tgTCR) clonotypes were identified by expression of TRAV9-1/TRBV19 gene segments and the TCRβ CDR3 amino acid sequence, including those clonotypes with missing TCRα chain.

#### RNA velocity analysis

RNA velocity analysis^53^ was performed using the scVelo (v.0.2.4) package [10]. RNA velocity was estimated by distinguishing unspliced and spliced mRNAs using the velocyto package (v.0.17) (https://velocyto.org/velocyto.py/^54^). Data were subclustered on T_reg_ cell, filtering out the subsets with fewer than ten cells per gut region (that is, excluding eTregs_MLN, eTregs_CP, cTregs_LA, cTregs_LP and Prolif-Tregs_CP). The dataset was then merged with the velocyto output (merged loom files) and pre-processed for detection of minimum number of counts, filtering and normalization (scvelo.pp.filter_and_normalize). The functions scvelo.pp.moments, scvelo.tl.velocity and scvelo.tl.velocity_graph were used to compute velocities using the stochastic mode in scVelo. The function scvelo.pl.velocity_embedding_stream was used to project the velocity information onto the UMAP. To test which genes have cluster-specific differential velocity expression and visualize selected genes, the functions scvelo.tl.rank_velocity_genes and scvelo.pl.velocity were applied. Velocity pseudotime was calculate with the function scvelo.tl.velocity_pseudotime.

#### Cell-type scoring

A list of mouse genes involved in the MHCII complex (Supplementary Table 2) was used for surface MHCII scoring. Cells were scored using the scanpy.tl.score_genes function according to the expression values of all genes.

#### Cell–cell communication analysis

The CellPhoneDB^55,56^ Python package (v.3 .0) was used to infer putative cell–cell interactions. The scRNA-seq dataset was split by gut region and cell types with <30 cells in a given region were filtered out. Human-mouse orthologue genes were retrieved using the biomaRt package^57^, and only one-to-one orthologous genes were considered. CellPhoneDB was applied on the normalized raw counts and fine cell-type annotations of myeloid, T cells and ILCs from LA and LP (separately for each gut region), using default parameters. To identify the most relevant interactions, specific interactions of T_reg_ cells with myeloid cells and T cells/ILCs were selected and filtered for the ligand–receptor pairs that were significant (*P* ≤ 0.01) and ‘curated’. The selected interactions were plotted as expression of both ligands and receptors in relevant cell types. The ktplots R package (https://github.com/zktuong/ktplots/tree/plot_cpdb3; 10.5281/zenodo.5717923) was used to visualize the significant interactions per cell-type pair using a chord diagram.

### Fresh frozen Visium sample preparation

Caecum and proximal colon tissue from *Hh-*infected and *Hh*/anti-IL10R treated mice were removed, cut longitudinally and cleaned of stool with cold phosphate buffered saline (PBS). The tissue was positioned luminal side up, and rolled into a Swiss roll from the caecum to the proximal colon. The tissue was placed into a histology plastic cassette and snap frozen for 1 min in dry-ice-cooled isopentane. The frozen tissue was embedded in OCT on dry ice and stored at −80 °C. The samples were selected based on tissue morphology and orientation (H&E-stained sections) and RNA integrity number, obtained using High sensitivity RNA ScreenTape system, (Agilent 2200 TapeStation). OCT blocks were sectioned at 10 μm thickness in a −20 °C cryostat (Leica CX3050S) at 10 μm, and transferred onto a 6 mm^2^ capture area on a Visium 10X Genomics slide. Visium spatial tissue optimization was performed, and an optimal permeabilization time of 24 min was selected. The Visium slides were processed according to manufacturer’s instructions, before fixing and staining H&E for imaging. H&E-stained slides were imaged at 40× on Hamamatsu NanoZoomer S60. After transcript capture, sequencing libraries were prepared according to the 10X Genomics Visium Spatial Transcriptomic protocol and sequenced on the Illumina Novaseq 600 system.

### Visium spatial transcriptomics data analysis

#### 10x Genomics Visium sequencing data processing

After sequencing, 10x Genomics Visium spatial samples were aligned to the mouse transcriptome mm10 2020-A reference (as the scRNA-seq samples) using 10x Genomics SpaceRanger version 2.0.0. and exonic reads were used to produce mRNA count matrices for each sample. SpaceRanger was also used to align paired histology images with mRNA capture spot positions on the Visium slides. A custom image-processing pipeline was used for alignment of Visium slides and identification of the spots contained in the tissue, as described in ref. ^58^. Spots with fewer than 500 UMI counts, and more than 15% mitochondrial genes were removed from the analysis. Data from different samples were concatenated and SCVI was used for batch correction^59^.

#### Spatial mapping of cell types using cell2location

To spatially map intestinal cell types defined by single-cell transcriptomics in the Visium data we used cell2location^41^. First, to obtain a complete single-cell reference of cell types and cell states in the mouse intestine we integrated our NICHE-seq data with 3 publicly available datasets of intestinal epithelial cells^30^, immune cells^15^ and enteric nervous system^27^ (Fig. 5a). Redundant cell annotations across different datasets were harmonized and curated manually. This scRNA-seq reference (untransformed and unnormalized mRNA counts) was then used in the cell2location pipeline, as described in detail previously^43^. In brief, reference signatures of cell states (63 cell populations) were estimated using a negative binomial regression model provided in the cell2ocation package. The inferred reference cell-state signatures were used for cell2location mapping that estimates the abundance of each cell state in each Visium spot by decomposing spot mRNA counts. The cell2location spatial mapping was done separately for *Hh* and *Hh*/anti-IL10R sections. The paired H&E images were used to determine the average number of cells per spot in the tissue (set to 30) and used as a hyperparameter in the cell2location pipeline. Cell-state proportions in each Visium spot were calculated based on the estimated cell-state abundances.

Two methods were used to identify cellular microenvironments in the tissue: manual annotation and conventional NMF analysis. Regions for manual annotation were defined based on H and E images. LA were defined by cellular density, whereas LP regions included histologically distinct areas both proximal and distal to the LA. NMF, implemented in the cell2location pipeline, was performed on cell abundance results by cell2location on each condition separately (Hh and *Hh*/anti-IL10R). The NMF model was trained for a range of factors and tissue zones (number of factors: n_fact) *N* = {5,…,30} and the decomposition into 18 factors was selected as a balance between segmenting relevant tissue zones (muscle compartment, lymphoid structures, lymphatics) and over-separating known zones into several distinct factors (Extended Data Fig. 8c).

#### Cell-state spatial enrichment analysis

Spots containing lymphoid aggregates and adjacent LP were manually annotated using the paired histology images of the spatial data in the 10x Genomics Loupe software. Cell-state proportions per spot were calculated based on the estimated abundances from cell2location and cell-state enrichments (odds ratio) in each manually annotated region were calculated as described^60^. In brief, the odds of target cell-state proportions were divided by the odds of the other cell-state proportions. Odds of cell proportions were calculated as the ratio of cell proportion in the spots of a structure of interest to that in the other spots. Statistical significance was obtained by chi-square analysis (scipy.stats.chi2_contingency) and the *P* value was corrected with the Benjamini–Hochberg method.

#### Spatial co-occurrence analysis of cell types

We quantified the degree of co-occurrence between cell types on the basis of their proportions inferred by cell2location. Specifically, since each cell type had an estimated abundance distribution across spatial spots, we calculated the Pearson correlation coefficient for any two given cell types as their co-occurrence rate. This calculation was conducted for each sample separately. Next, the log_2_ ratio between four *Hh*-only (control) samples and four *Hh*/anti-IL10R samples was defined as their fold change in co-occurrence rate and the significance (that is, *P* value) was assessed by a two-sided Student’s *t*-test.

##### Spatial ligand–receptor analysis

Ligand–receptor analysis on Visium data was performed using the Cell2location cell-type abundances and the stLearn package^61^ (https://github.com/BiomedicalMachineLearning/stLearn). In short, the connectomeDB2020_lit database for mouse was used as a reference of candidate ligand–receptor pairs. The st.tl.cci.run function was used to calculate the significant spots of ligand–receptor interactions within spot mode (distance = None), filtering out any ligand–receptor pairs with no scores for less than 20 spots, and using 10,000 random pairs (n_pairs). *P* values were corrected with the st.tl.cci.adj_pvals function using false discovery rate, Benjamini–Hochberg (adj_method = ‘fdr_bh’) adjusting by the number of spots tested per ligand–receptor pair (correct_axis = ‘spot’). Spot *P* values were displayed for particular ligand–receptor pairs (‘Ccl8_Ccr2’ and ‘Cxcl9_Cxcr3’) in the spatial context using the function st.pl.lr_result_plot.

### Statistical analysis

Statistical analysis was performed using Prism 8 (GraphPad). *t*-Tests were used to compare two unpaired samples. For more than two groups, the ordinary one-way ANOVA was used. No samples were excluded from analysis. Mean with standard deviation shown unless otherwise indicated. Differences were considered statistically significant when *P* ≤ 0.05. Significance is indicated as *P ≤ 0.05, **P ≤ 0.01, ***P ≤ 0.001 and ****P ≤ 0.0001. Technical replicates were processed and analyses on the same day. Biological replicates are from independent experiments.

### Reporting summary

Further information on research design is available in the Nature Portfolio Reporting Summary linked to this article.

## Online content

Any methods, additional references, Nature Portfolio reporting summaries, source data, extended data, supplementary information, acknowledgements, peer review information; details of author contributions and competing interests; and statements of data and code availability are available at 10.1038/s41586-024-07251-0.

### Supplementary information

Supplementary Figs. 1–5 Detailed information regarding single-cell bioinformatics analysis workflow, cell type determination, and spatial transcriptomics analysis.

Reporting Summary

Supplementary Table 1 Differentially expressed genes for each cluster, grouped by cell lineages. Statistical test used was Wilcoxon rank-sum implemented in Scanpy v.1.7. P value correction was performed using the Benjamini–Hochberg method.

Supplementary Table 2 Compiled gene lists used for the different scores.

Supplementary Video 1 Rotation of a 3D volume of caecum imaged with a two-photon microscope shows a LA highlighted by green T_reg_ cells with TCR^*Hh*^*CD2*^DsRed^Nur77^GFP^ cells with red cytoplasm and green nuclei.

Supplementary Video 2 Time lapse of a TCR^*Hh*^*CD2*^DsRed^Nur77^GFP^ cell in the caecum LP demonstrating motility through the tissue.

Supplementary Video 3 Time lapse of TCR^*Hh*^*CD2*^DsRed^*Il10*^*GFP*^ T cells in the caecum LP demonstrating motility through the tissue.

Supplementary Video 4 Time lapse of IL-10+ TCR^*Hh*^ cell interacting with CD206^+^ macrophages in the caecum LP.

### Source data

Source Data Figs. 1–5 and Source Data Extended Data Figs. 2–4 and 7.

## Data Availability

Sequencing data for scRNA-seq, scTCR-seq and Visium data are available at the European Nucleotide Archive under accession PRJEB57700. Processed data for scRNA-seq and Visium experiments are available for browsing gene expression and downloading at https://treg-gut-niches.cellgeni.sanger.ac.uk/.  Source data are provided with this paper.

## Code availability

Code scripts and notebooks for analysis in the manuscript are available at https://github.com/Teichlab/treg-gut-niches/.
