# Supplementary material for: Immune microniches shape intestinal Treg function
Source: Nature. 2024 Apr 3;628(8009):854–62. doi: 10.1038/s41586-024-07251-0 (PMC11041794; doi:10.1038/s41586-024-07251-0)
Supplement: Supplementary file 1 — Detailed information regarding single-cell bioinformatics analysis workflow, cell type determination, and spatial transcriptomics analysis. [file 41586_2024_7251_MOESM1_ESM.pdf]

nature portfolio

https://doi.org/10.1038/s41586-024-07251-0

Supplementary information

Immune microniches shape intestinal T_{reg} function

In the format provided by the authors and unedited

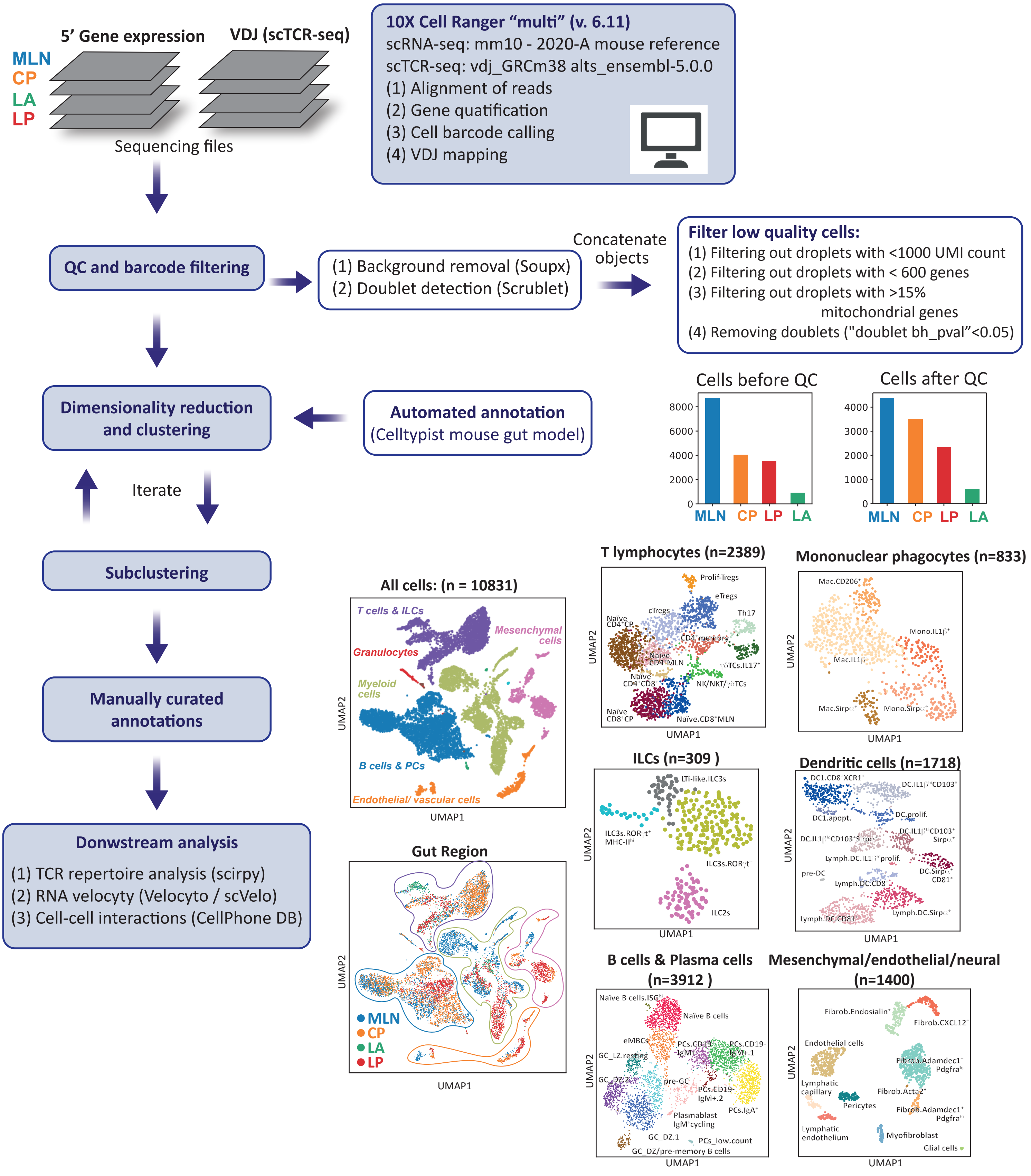

Supplementary Data 1.
Detailed single-cell analysis workflow.

a

Datasets used to build the CellTypist model for automatic annotation.

Dataset	# cells	# genes	Description	# cell types
Xu et al., 2019	58,067	19,187	**Immune cells** from the lamina propria (LP) and Peyer's patches (PP)	41
Kalucka et al., 2020	3,806	9,702	**Endothelial cells** from the small and large intestine	8
Haber et al., 2017	51,164	25,064	**Epithelial cells** from the small intestine	39
Drokhlyansky et al., 2020	10,226	24,753	**Neurons and glia** from the colon and ileum	26
Tabula Muris	10,198	22,786	**Total cells** from mouse large intestine	8
Corbin et al., 2020	5,886	25,168	**Mononuclear phagocytes** from colonic LP	10
Biton et al., 2018	28,462	24,507	**Epithelial** cells and **immune cells** from the the LP of the small intestine	23
Grenov et al., 2022	11,227	14,939	**B cells** from spleen	6

b

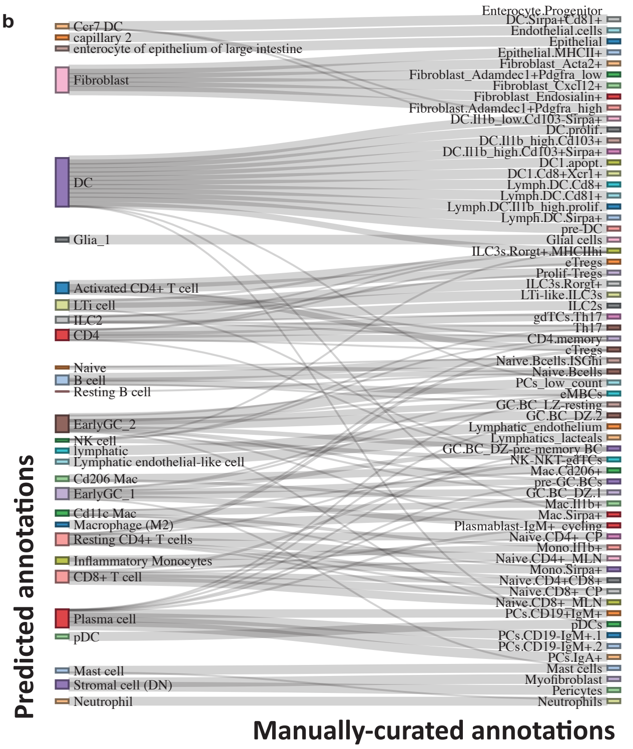

c

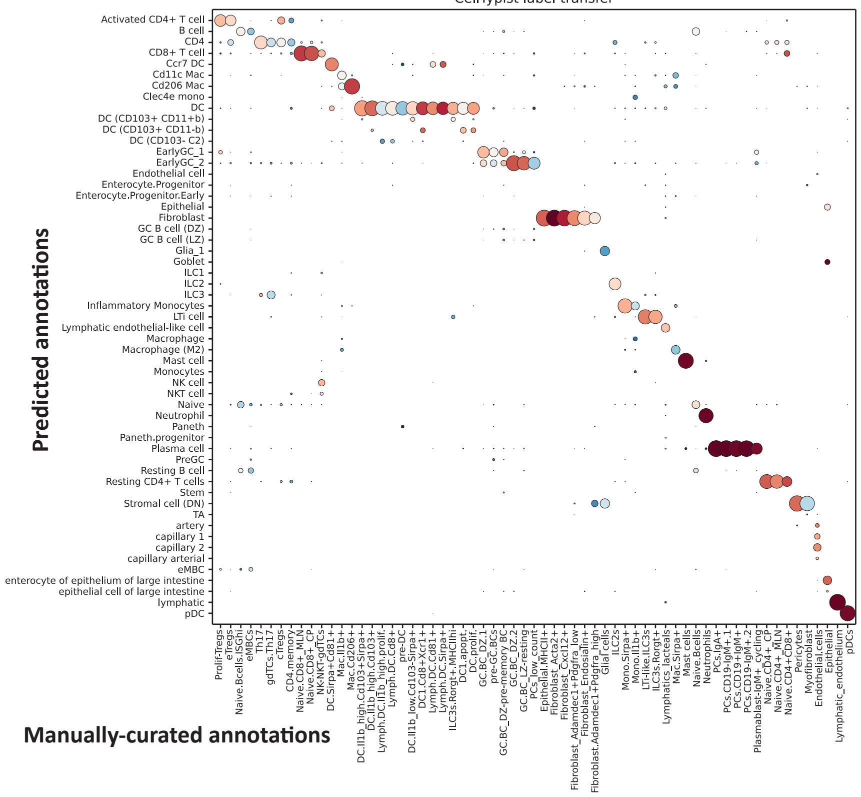

d

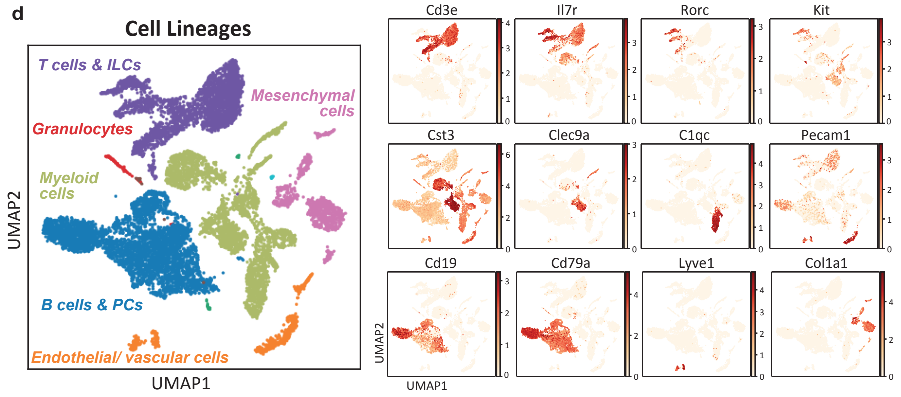

e

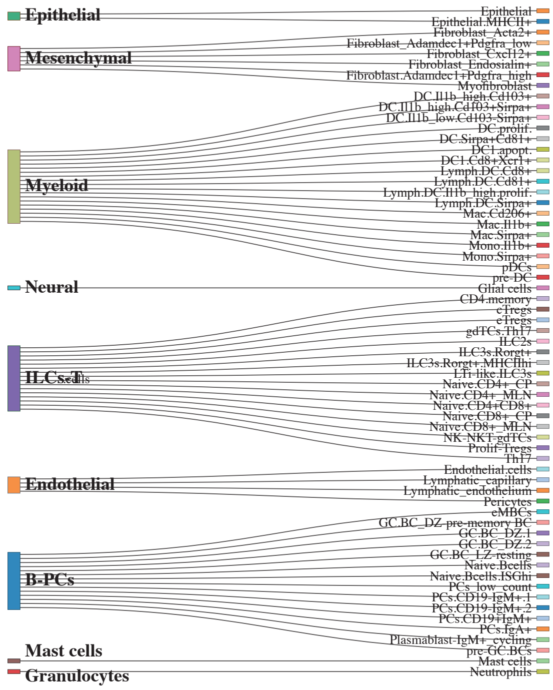

Supplementary Data 2.

a. Model for annotation of cell lineages. Information regarding the datasets used to build the CellTypist model for annotation of murine intestinal cells. **b.** Sankey plot showing the correspondence between predicted and manually curated cell-type annotations.
c. Dot plot showing the cross-validation by transferring the cell-type labels from our model (rows) to our dataset (columns), after manual-curation of the cell-type annotations (bottom). For each column (cell types from the CellTypist training datasets), the size of the dot corresponds to the proportion of cells assigned to a given cell type of the model, and the colour denotes the average probabilities calculated with CellTypist.
d. UMAP visualization of the total cells coloured by cell lineage (left). UMAP overlay of key genes defining T cells, innate lymphoid cells, B cells, myeloid cells, endothelial and stromal cells (right).
e. Sankey plot depicting the cell types belonging to each cell lineage.

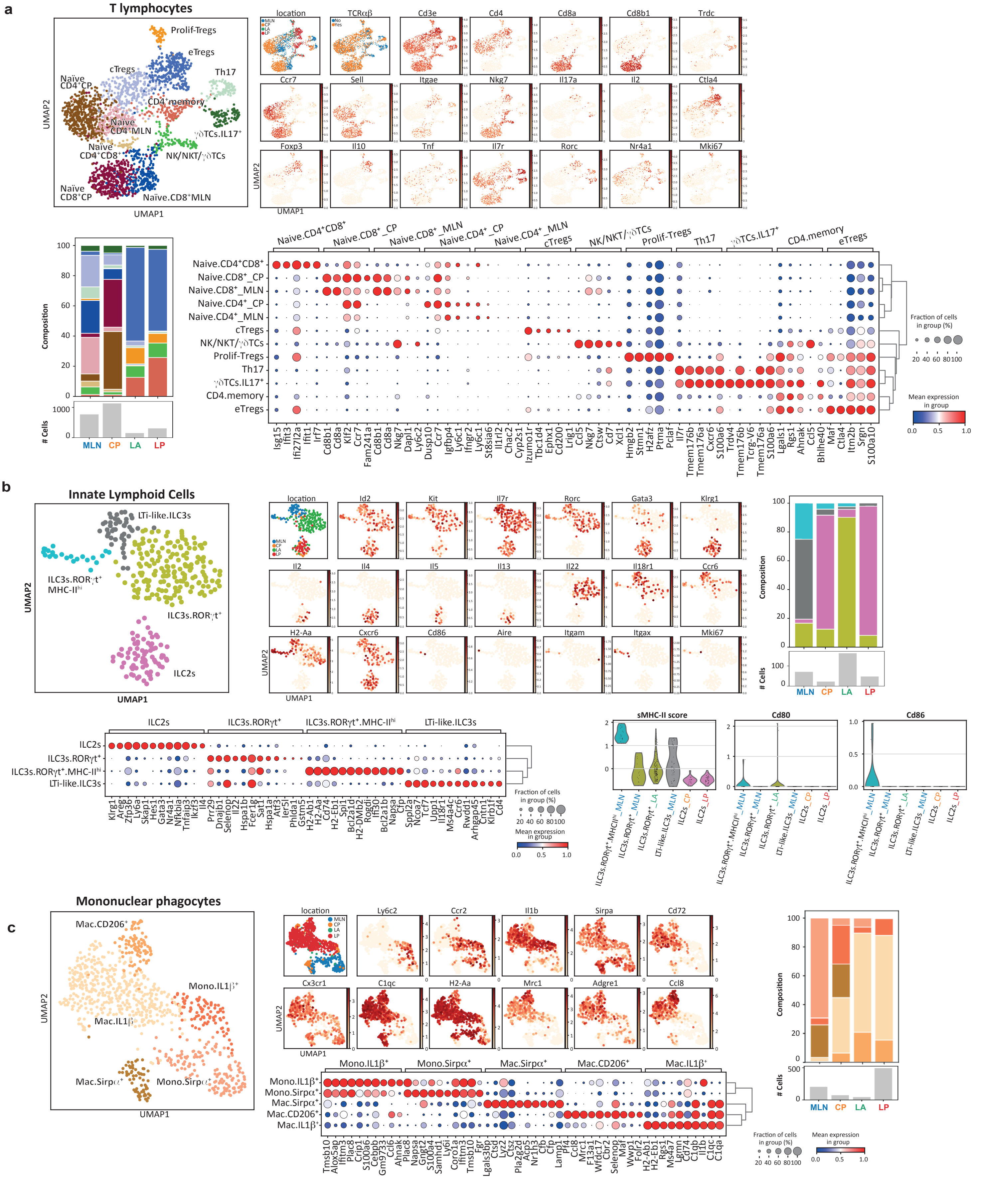

Supplementary Data 3. Marker genes defining cell T, ILC and mononuclear phagocyte lineages

a. UMAP visualisation of T cells across all locations (top left), overlay of selected marker genes on the UMAP plot (top right), bar chart showing regional distribution of subsets and total cell numbers (bottom left), and differential expression of T cell lineage defining marker genes (bottom right).

b. UMAP visualisation of ILC cells across all locations (top left), overlay of selected marker genes on the UMAP plot (top centre), bar chart showing regional distribution of subsets and total cell numbers (top right), and differential expression of ILC cell lineage defining marker genes (bottom left). Violin plots of MHC-II expression score in the ILC subsets at the indicated locations (bottom right).

c. UMAP visualisation of mononuclear phagocytes across all locations (top left), overlay of selected marker genes on the UMAP plot (top centre), bar chart showing regional distribution of subsets and total cell numbers (top right), and differential expression of mononuclear phagocytes cell lineage defining marker genes (bottom centre).

Supplementary data 4

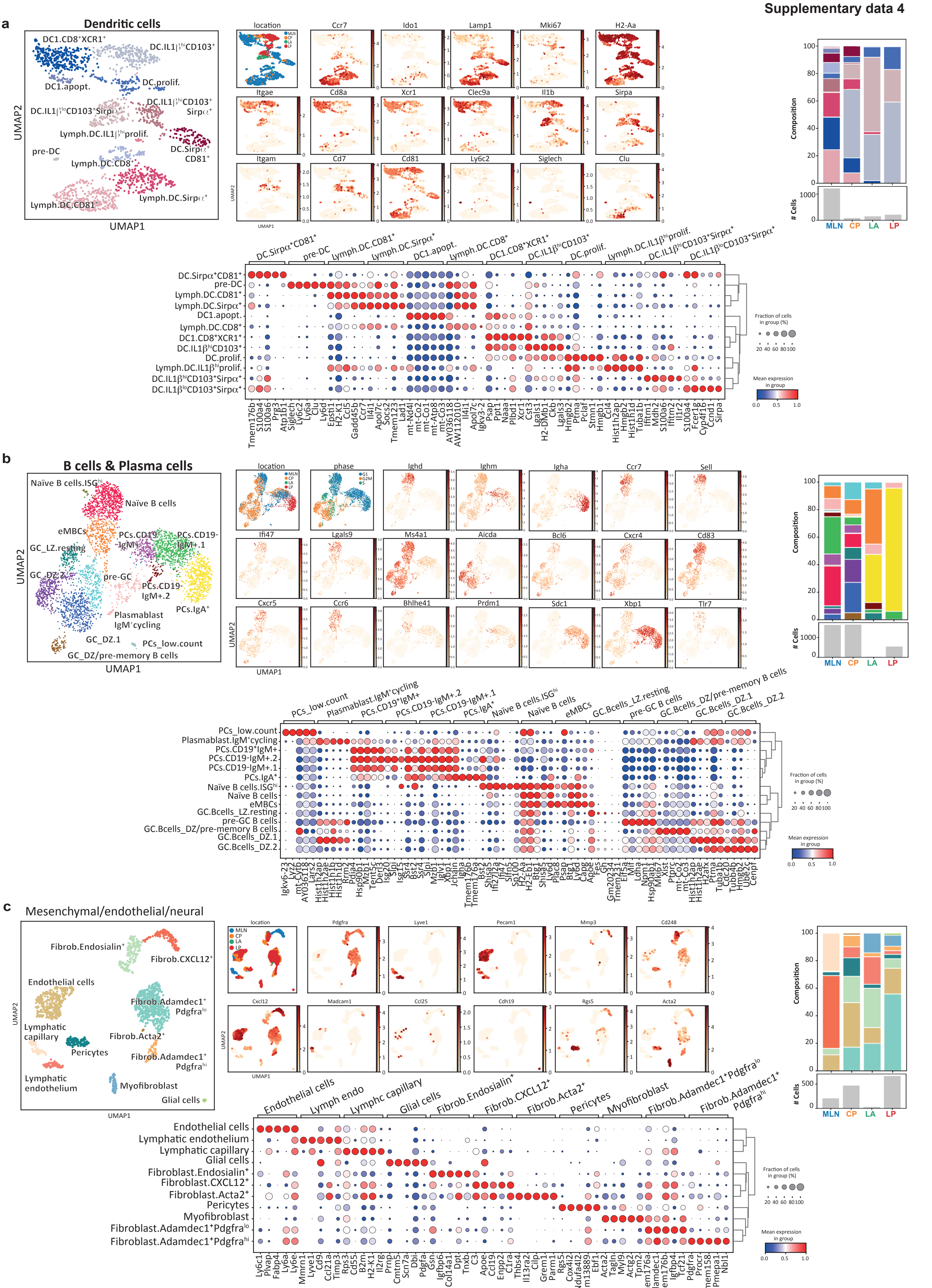

Supplementary Data 4. Marker genes defining dendritic, B and mesenchymal, endothelial and neural cell lineages
a-c. UMAP visualisation across all locations (top left), overlay of selected markers on the UMAP plot (top middle), bar chart showing regional distribution of subsets and total cell numbers (top right), and differential expression of marker genes defining subsets of the following lineages (bottom): a. Dendritic cells, b. B cells and Plasma cells, c. Mesenchymal, endothelial, and neural.

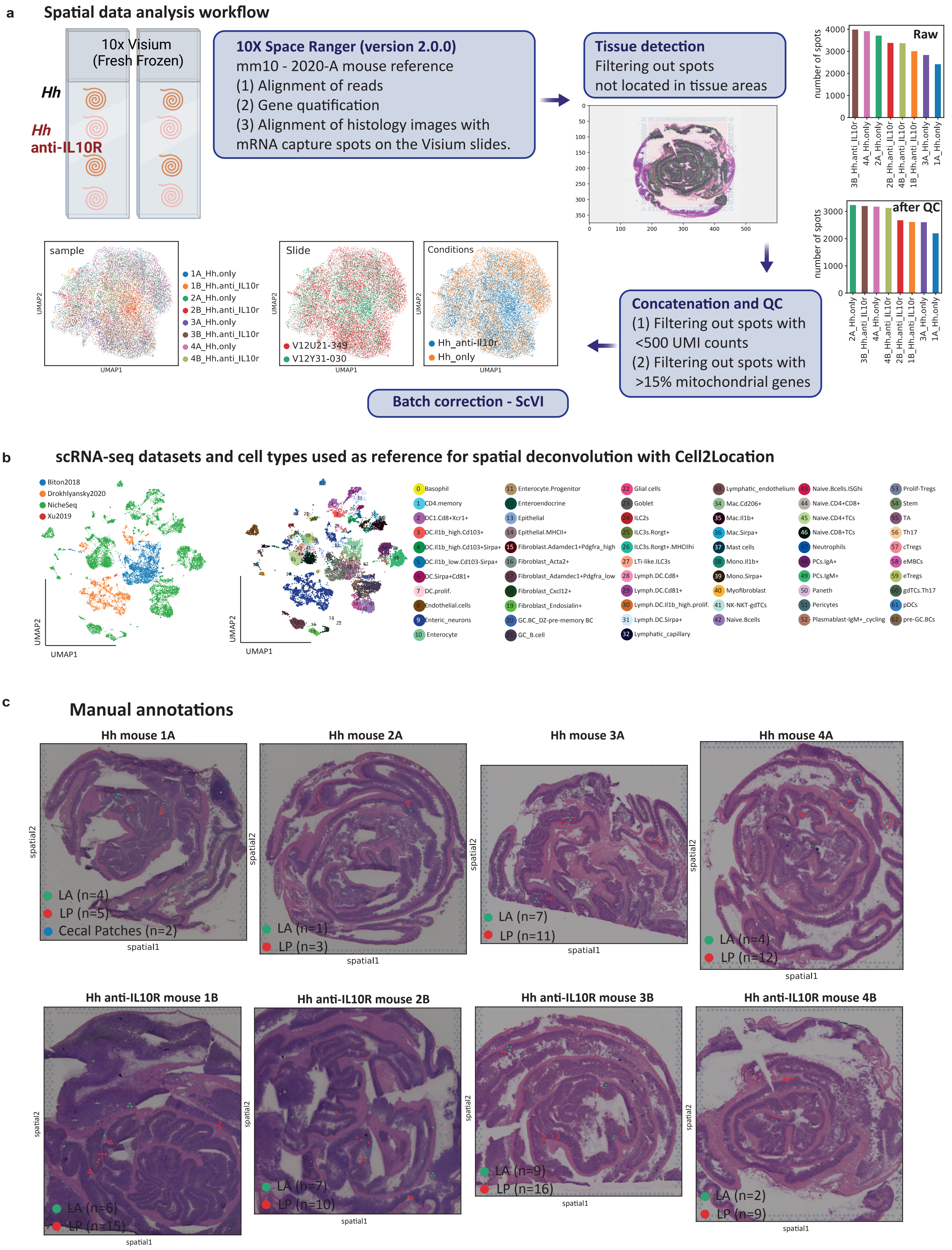

Supplementary Data 5.
a. Detailed spatial data analysis workflow.
b. scRNA-seq datasets and cell types used as reference for spatial deconvolution with Cell2Location
c. Tissue sections from 4 Hh (top) and 4 Hh/anti-IL10R (bottom) stained with H&E with manually annotated LA, LP, and caecal patch niches.
