# Supplementary material for: Immune microniches shape intestinal Treg function
Source: Nature. 2024 Apr 3;628(8009):854–62. doi: 10.1038/s41586-024-07251-0 (PMC11041794; doi:10.1038/s41586-024-07251-0)
Supplement: Supplementary file 2 — Reporting Summary [file 41586_2024_7251_MOESM2_ESM.pdf]

nature portfolio

Corresponding author(s): Fiona Powrie and Emily Thornton

Last updated by author(s): 02/02/2024

Reporting Summary

Nature Portfolio wishes to improve the reproducibility of the work that we publish. This form provides structure for consistency and transparency in reporting. For further information on Nature Portfolio policies, see our Editorial Policies and the Editorial Policy Checklist.

Statistics

For all statistical analyses, confirm that the following items are present in the figure legend, table legend, main text, or Methods section.

n/a	Confirmed	
☐	☒	The exact sample size (*n*) for each experimental group/condition, given as a discrete number and unit of measurement
☐	☒	A statement on whether measurements were taken from distinct samples or whether the same sample was measured repeatedly
☐	☒	The statistical test(s) used AND whether they are one- or two-sided<br>*Only common tests should be described solely by name; describe more complex techniques in the Methods section.*
☒	☐	A description of all covariates tested
☐	☒	A description of any assumptions or corrections, such as tests of normality and adjustment for multiple comparisons
☐	☒	A full description of the statistical parameters including central tendency (e.g. means) or other basic estimates (e.g. regression coefficient) AND variation (e.g. standard deviation) or associated estimates of uncertainty (e.g. confidence intervals)
☐	☒	For null hypothesis testing, the test statistic (e.g. *F*, *t*, *r*) with confidence intervals, effect sizes, degrees of freedom and *P* value noted<br>*Give P values as exact values whenever suitable.*
☒	☐	For Bayesian analysis, information on the choice of priors and Markov chain Monte Carlo settings
☒	☐	For hierarchical and complex designs, identification of the appropriate level for tests and full reporting of outcomes
☒	☐	Estimates of effect sizes (e.g. Cohen's *d*, Pearson's *r*), indicating how they were calculated

Our web collection on statistics for biologists contains articles on many of the points above.

Software and code

Policy information about availability of computer code

Data collection	No commercial or custom software/code was used to collect the data in this study.
Data analysis	All algorithms or software used to analyse the data are publicly available.<br>Code for the data analysis will be available at publication.

For manuscripts utilizing custom algorithms or software that are central to the research but not yet described in published literature, software must be made available to editors and reviewers. We strongly encourage code deposition in a community repository (e.g. GitHub). See the Nature Portfolio guidelines for submitting code & software for further information.

Data

Policy information about availability of data

All manuscripts must include a data availability statement. This statement should provide the following information, where applicable:

- Accession codes, unique identifiers, or web links for publicly available datasets
- A description of any restrictions on data availability
- For clinical datasets or third party data, please ensure that the statement adheres to our policy

Public datasets used in this study were described in previous publications (PMID: 32888429, 29144463, 32059779, 32669714, 31618654, 30392957, 32444476, 35508130).

Data availability

March 2021

Sequencing data for scRNA-seq, scTCR-seq and Visium data is available at https://www.ebi.ac.uk/ena/browser/view/PRJEB57700. Processed data of scRNA-seq and Visium is available for browsing gene expression and downloading in https://treg-gut-niches.cellgeni.sanger.ac.uk/. Code scripts and notebooks for analysis in the manuscript are available at https://github.com/Teichlab/treg-gut-niches/

Field-specific reporting

Please select the one below that is the best fit for your research. If you are not sure, read the appropriate sections before making your selection.

☒ Life sciences ☐ Behavioural & social sciences ☐ Ecological, evolutionary & environmental sciences

For a reference copy of the document with all sections, see nature.com/documents/nr-reporting-summary-flat.pdf

Life sciences study design

All studies must disclose on these points even when the disclosure is negative.

Sample size	No statistical methods were used to predetermine sample size. Sample sizes were based on previous similarly designed experiments from our research group. The spatial transcriptomics experiment included 4 mice per group to balance statistical power with cost. For other experiments we aimed for a minimum of 5 mice per experimental group. Exact mouse numbers for each experiment are included in the figure legends.
Data exclusions	No data were excluded from analysis
Replication	All data are representative of at least two independent experiments, with reliable replication of results.
Randomization	Mice were assigned to different experimental groups at random. Mice were co-housed and littermate when possible. Each cage contained all treatment conditions.
Blinding	Animal studies were not blinded.<br>Histopathology scoring was conducted by two independent assessors, one of whom was blinded.

Reporting for specific materials, systems and methods

We require information from authors about some types of materials, experimental systems and methods used in many studies. Here, indicate whether each material, system or method listed is relevant to your study. If you are not sure if a list item applies to your research, read the appropriate section before selecting a response.

Materials & experimental systems

n/a	Involved in the study	
☐	☒	Antibodies
☒	☐	Eukaryotic cell lines
☒	☐	Palaeontology and archaeology
☐	☒	Animals and other organisms
☒	☐	Human research participants
☒	☐	Clinical data
☒	☐	Dual use research of concern

Methods

n/a	Involved in the study	
☒	☐	ChIP-seq
☐	☒	Flow cytometry
☒	☐	MRI-based neuroimaging

Antibodies

Antibodies used	Antibodies for flow cytometry:<br>anti-mouse CD11c N418 PerCP/Cyanine5.5/ BV605 Biolegend<br>anti-mouse CD11b M1/70 PerCP/Cyanine5.5/BV605 eBioscience<br>anti-mouse CD4 RM4-5 BV785 Biolegend<br>anti-mouse TCRb H57-597 AF700 Biolegend<br>anti-mouse CD45.1 A20 BV650/ PerCP/Cyanine5.5/APC Biolegend<br>anti-mouse CD45.2 104 APC/BV605 Biolegend<br>anti-mouse CD3 145-2C11 FITC/ PerCP/Cyanine5.5 Biolegend<br>anti-mouse B220 RA3-6B2 PerCP/Cyanine5.5/BV605 Biolegend<br>anti-mouse CD44 IM7 AF700 Biolegend<br>anti-mouse CD45 30-F11 BV605 Biolegend<br>anti-mouse TCRVb6 RR4-7 APC Biolegend<br>anti-mouse CD25 Ebio3c7 eFluor450 eBioscience<br>anti-mouse CD62L MEL-14 PE/BV510 eBioscience<br>anti-human CD2 TS1/8 BV421 Biolegend

anti-mouse Ki-67 SolA15 FITC, Thermo Fisher Scientific
anti-mouse Foxp3 FJK-16S PE-eFluor 610 Thermo Fisher Scientific
anti-mouse Rorgt Q31-378 BV786 Thermo Fisher Scientific
anti-mouse c-Maf T54-853 PE BD Biosciences
anti-mouse TCF1/TCF7 C63D9 Pacific Blue Cell Signaling Technology

Antibodies for immunofluorescent staining:
anti-mouse CD4 RM4-5 PE Biolegend
anti-mouse B220 RA3-6B2 FITC Biolegend
anti-mouse MHCII M5/114.15.2 APC Biolegend
anti-mouse CD172a (Sirpa) P84 A488 Biolegend
anti-mouse CD11c N418 A647 Biolegend
anti-mouse CD206 C068C2 A594 and FITC Biolegend

Validation	All antibodies were purchased from Biolegend, eBioscience, Thermo Fisher Scientific or Cell Signaling Technology<br>All antibodies were validated by the manufacturers and in previously published data. For immunofluorescence staining, single stains and isotype controls were used to validate staining and acquisition conditions.

Animals and other organisms

Policy information about studies involving animals; ARRIVE guidelines recommended for reporting animal research

Laboratory animals	B6.Foxp3GFP , B6Rag1-/-, B6.dsRedhCD2, B6.Foxp3hCD2IL-10GFP, UBC-PA-GFP, B6.Nur77GFP, LTa-/- and TCRHh were on a C57BL/6 background.<br><br>Experimental and control groups were matched for age and gender. Mice were between 6 to 12 weeks old.
Wild animals	This study did not involve wild animals
Field-collected samples	This study did not involve field-collected samples
Ethics oversight	All experiments were conducted in accordance with the UK Scientific Procedures Act of 1986, and by persons holding a personal license, working under a project license authorised by the UK Home Office.

Note that full information on the approval of the study protocol must also be provided in the manuscript.

Flow Cytometry

Plots

Confirm that:

☒ The axis labels state the marker and fluorochrome used (e.g. CD4-FITC).

☒ The axis scales are clearly visible. Include numbers along axes only for bottom left plot of group (a 'group' is an analysis of identical markers).

☒ All plots are contour plots with outliers or pseudocolor plots.

☒ A numerical value for number of cells or percentage (with statistics) is provided.

Methodology

Sample preparation	Isolation of intestinal tissue and lymphoid cells for flow cytometry are detailed in the methods section of the manuscript.<br><br>Intestinal tissue were washed twice in RPMI (Sigma Aldrich)/10%FCS/5mM EDTA at 37oC with agitation for 25 mins to remove epithelial cells. Caecal patch and OLS were removed under x40 brightfield microscopy using a scalpel and a 16G needle and syringe respectively. Remaining colon and caecum tissue, OLS and caecal patches were digested for 40 minutes at 37oC with agitation in RPMI/10%FCS15mM Hepes with 100U/ml collagenase VIII (Sigma Aldrich) and 20mg/ml DNase I (Sigma Aldrich). Leukocytes from colon/caecum tissue were recovered at the interface of a 40/70% Percoll gradient (Fisher Scientific).<br><br>Spleens and MLNs were mechanically disrupted, and splenic red blood cells lysed with ACK lysis buffer.<br><br>Peripheral blood was collected by cardiac puncture and red cells lysed with ACK lysis buffer.<br><br>For spatial transcriptomics analysis, caecum and proximal colon tissue were prepared as detailed in the methods section of the manuscript.

Instrument	LSRII BD LSRFortessa X20 for cell analysis<br>FACSAria III for cell sorting<br>Sequencing performed on the Illumina Novoseq 600 system for spatial transcriptomics analysis
Software	BD FACSDiva (BD Bioscience) and FlowJo v10.8 (Treestar)
Cell population abundance	Live GFP+ single cells were collected by photo-activated cell sorting at the following locations:<br>mLN: 7857<br>Caecal patch: 3905<br>ILF: 918<br>lamina propria: 3231<br><br>For adoptive transfer of naive HH7-2tg, the sorted T cells had purities of greater than 98%
Gating strategy	SSC-A vs FSC-A was used to gate on lymphocytes. FSC-H vs FSC-A was used to gate on singlets.<br><br>TCRHh cells were gated as live, single cells that were CD11b- CD11c- CD4+ TCRbeta+ CD45.1+<br>Host cells were gated as live, single cells that were CD11b- CD11c- CD4+ TCRbeta+ CD45.2+<br><br>Cell sorting for naive TCRHh for adoptive transfer were gated on live single cells that were CD45+ CD3+ CD11c- CD11b- B220- CD4+ CD62L+ CD44- TCRVb6+<br><br>Positive populations were distinct from negative populations, and fluorescence minus one (FMO) samples were used if these two populations were not clearly separated. Fluorophores were chosen to minimise spectral overlap.

☒ Tick this box to confirm that a figure exemplifying the gating strategy is provided in the Supplementary Information.
